# Polymer Crosslinked Activated Carbon Pellets for Dye Adsorption

**DOI:** 10.3390/ma19010155

**Published:** 2026-01-02

**Authors:** Muhammad Hadi, Sungho Yoon

**Affiliations:** Department of Chemistry, Chung Ang University, 84 Heukseok-ro, Dongjak-gu, Seoul 06974, Republic of Korea

**Keywords:** pelletization, crosslinked polyvinyl alcohol–diglycidyl ether of bisphenol a matrix, adsorption, disintegration, methylene blue

## Abstract

The use of activated carbon (AC) in environmental applications, particularly for water and air purification, is highly valued due to its excellent microstructural and adsorption properties. However, its powdered form presents significant challenges in industrial applications, such as difficulty in handling and potential environmental risks due to its tendency to disperse easily. To overcome these issues, converting activated carbon into a more industrially viable form, such as pellets, is crucial. In this study, pelletizing AC within a crosslinked polyvinyl alcohol–diglycidyl ether of bisphenol A (PVA–DGEBA) matrix enabled the production of structurally stable cylindrical pellets through the formation of a robust three-dimensional polymeric network. This approach required minimal binder usage and facilitated processing at relatively low temperatures, effectively overcoming common disintegration issues associated with traditional pelletization methods reliant on linear polymer binders and compression-based techniques. The resulting pellets exhibited methylene blue (MB) adsorption (q max ~14.8 mg/g of pellet), which is about 50% of the initial AC’s adsorption capability, and retained structural integrity across multiple aqueous cycles. They also remained stable in methanol, ethanol and acetone by showing no observable disintegration, which highlights their excellent stability. Comprehensive characterizations, including hardness tests, swelling behavior, and various structural evaluations, revealed a mechanical strength of 3.37 ± 0.46 MPa and an adsorption volume of ~250 cm^3^/g through Brunauer–Emmett–Teller analysis, confirming effective crosslinking and the adsorption capabilities of the pellets. This eco-friendly and stable pelletization strategy demonstrated great potential for low-temperature pelletizing of AC, ensuring advanced applications in wastewater treatment even under pressurized conditions, presenting a significant improvement over the traditional method.

## 1. Introduction

Activated carbon (AC) has been used in various applications such as CO_2_ capture [1] and wastewater treatment [2,3], owing to its exceptional adsorption capacity, high porosity, and distinct microporous and mesoporous structure [4,5]. These properties make it highly effective for various adsorption applications. However, in its powdered form, AC presents significant challenges in handling, recovery, and disposal in large-scale applications [6,7]. Despite this, its low bulk density leads to increased volume requirements, resulting in inefficient storage and transport. The fine-powdered particles tend to disperse easily, necessitating complex filtration or separation processes that complicate recovery, often resulting in material loss. Additionally, issues such as prolonged settling, hydraulic pressure buildup, and increased flow resistance further hinder operational efficiency. While magnetization has been investigated as a recovery method, it can undesirably alter surface properties and reduce the adsorption efficiency of AC [8]. In contrast, a more scalable and efficient approach involves transforming powdered AC into structured morphologies such as cylinders, spheres, or rings. These engineered forms improve material handling, reduce flow resistance, and retain the inherent porosity crucial for adsorption efficiency, making them ideal for industrial applications including adsorption, filtration, and wastewater treatment [9].

Pellet fabrication traditionally involves extruding fine AC powder with a binder to create dense, compressed structures, followed by high-temperature sintering [10,11,12,13]. A wide range of binders have been studied in pellet synthesis, including cellulose derivatives [14,15,16,17], chitosan [18], Eudragit L30 [19], Eudragit RS100 [20], polyvinyl acetate phthalate, Xyloglucan and xanthan gum [21], polyvinylpyrrolidone and guar gum [22], sodium alginate [16,23], polyacrylic acid, Avicel^®^ PH101 [24], Topical Starch [25], and Matrimid 5218 [26]. While these linear or chain-like binders are effective in holding the particles together, they typically depend on weak physical forces, resulting in pellets with limited structural integrity. These binders are widely used in the pharmaceutical industry for pellet formation through extrusion–spheronization techniques, often producing pellets that disintegrate easily [27], making them unsuitable for applications requiring durable pellet forms for environmental management. There are other methods to bind materials via polymers [28,29], including more stabilized approaches based on polymer crosslinking, which are specifically known for their chemical and environmental stability and improved resistance to disintegration. Examples include DGEBA with TETA [30] and Methyl Anhydride [31], PVA with GA (Glutaraldehyde) [32], PEI [33], citric acid [34], suberic acid, terephthalic acid [35], FDCA [36], succinic acid [37], and DGEBA [38]. However, these methods are predominantly applied in membrane fabrication or typically require high polymer binder contents (≥70%), which may block active sites and reduce porosity, ultimately compromising adsorption performance.

In this study, polyvinyl alcohol (PVA) was identified as an effective polymer matrix, with the diglycidyl ether of bisphenol A (DGEBA) serving as a crosslinker to form activated carbon (AC) pellets with 30 wt.% binder content. This was achieved through efficient epoxy–hydroxyl crosslinking and enhanced particle encapsulation, facilitated by PVA’s strong adhesive properties [39]. Moreover, the PVA-based binder system offers advantages of low cost and is considered one of the green and environmentally compatible binding system [40]. The resulting pellets exhibit remarkable resistance to disintegration after multiple methylene blue (MB) adsorption cycles and exposure to solvents such as methanol, ethanol, and acetone. They maintained impressive mechanical strength (3.37 ± 0.46 MPa) even when produced at lower pelletizing temperatures compared to traditional methods, and demonstrated enhanced thermal stability for the crosslinked matrix, as confirmed by thermogravimetric analysis (TGA). Fourier transform infrared (FTIR) spectroscopy and X-ray diffraction (XRD) analysis further confirmed the successful crosslinking within the polymer matrix, evidenced by the emergence of new ether peaks (~1250 cm^−1^) and a reduction in the hydroxyl peak (~3400–3600 cm^−1^), along with the disruption of PVA crystallinity via DGEBA addition to form a stable polymeric network, which encapsulated AC particles into robust pellets. This combination of properties positions the pellets as highly effective for adsorption applications, especially in industries such as textiles and apparel, where dye usage is prevalent. Additionally, this pelletizing approach offers a promising solution for materials that can only withstand temperatures up to 200 °C and are prone to microstructural collapse under high pressure, combining both environmental sustainability and industrial applicability.

## 2. Materials and Methods

### 2.1. Materials and Instrumentation

All chemicals were used as received. DMSO (Dimethyl Sulfoxide) (C_2_H_6_OS), Bisphenol A diglycidyl ether (C_21_H_24_O_4_), Polyvinyl Alcohol ([C_2_H_4_O]_n_), methylene blue (C_16_H_18_ClN_3_S), and AC were purchased from Sigma Aldrich (Burlington, MA, USA), and all these chemicals were of analytical grade. N_2_ (99.99%) was obtained from Sinyang Gas Industries, Cheongju-si, Republic of Korea.

Field emission scanning electron microscopy (FE-SEM) was conducted using an SIGMA 360 (Carl Zeiss) (Tokyo, Japan) at an accelerating voltage of 5.00 kV to observe the surface morphology of the samples. Nitrogen (N_2_) adsorption–desorption measurements were carried out at 77 K using a BELSORP mini II (BEL Japan, Osaka, Japan) after degassing the samples at 105 °C for 24 h to determine their textural properties. Infrared spectra were recorded with an NICOLET iS10 50 FTIR spectrometer (Thermo Scientific, Waltham, MA, USA) equipped with a Smart ATR-IR accessory to identify functional groups. X-ray diffraction (XRD) analysis was performed to investigate the crystallographic structure and phase composition of the materials. UV–visible absorption spectra were measured using a V-750 spectrophotometer (Jasco, Tokyo, Japan) to assess dye adsorption performance. Compressive strength tests were conducted with a JSV-H1000 hardness tester (AIKOH) (Parteck Corp., Seoul, Republic of Korea) to evaluate the mechanical integrity of the pellets. Thermogravimetric analysis (TGA) was carried out on a TGA N-1000 (Scinco, Seoul, Republic of Korea) to examine thermal stability and decomposition behavior. Prior to pellet formation, activated carbon was finely ground using a rotary roller ball miller (LK, Lab Korea, Namyangju-si, Republic of Korea) operated at 340 rpm and 80% load.

### 2.2. Experimental Method

**Figure 1:**
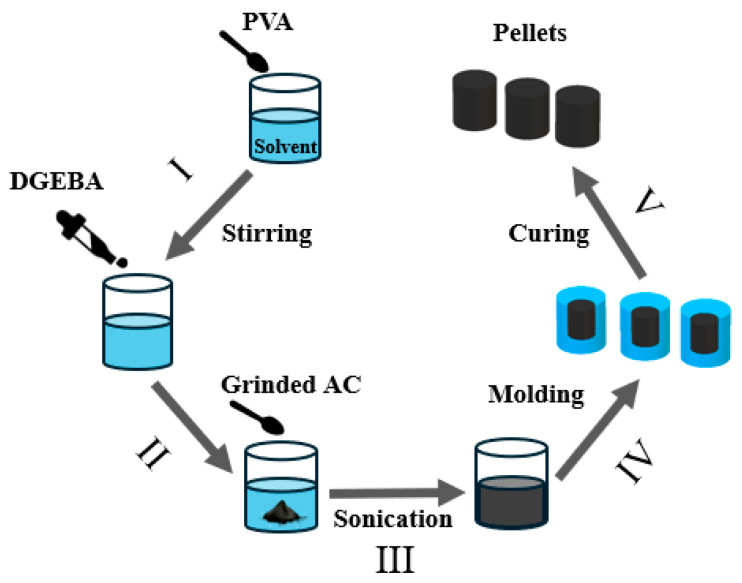
Schematic illustration of AC pelletization: (I) Mixing PVA with solvent, (II) Mixing DGEBA with PVA solution and adding ground AC, (III) Sonication for dispersion, (IV) Molding into pellet shape, and (V) Curing molds to form robust pellets.

To fabricate the AC pellets, Figure 1 shows the complete schematic fabrication process of pellets in which PVA was dissolved in DMSO at around 100 °C for 2 h until a clear and transparent solution was obtained. Next, DGEBA was added to the PVA solution, and the mixture was stirred at 80 °C for 1 h to form a homogeneous and transparent PVA-DGEBA blend. AC (500–800 μm) was ball milled for 12 h with 28 mm diameter zirconia balls at 340 rpm speed to reduce the size up to approximately 100 μm, which helps the binder blend penetrate easily and evenly in AC particles, and ultimately reinforces the pellet’s mechanical strength (see Figures S1 and S2 in the Supporting Information). The resulting powder was then gradually introduced into the blend while manually mixing the solution until a smooth and uniform paste was formed. To ensure homogeneity, the paste was further sonicated under heating conditions. It was then transferred into silicone molds and subjected to a stepwise curing process, following a predefined heating cycle from 80 °C to 190 °C for 24 h. After curing, the solidified pellets were removed from the molds and subjected to characterization. An additional experiment was conducted by immersing the prepared pellets (18 wt.%, 25 wt.%, and 30 wt.% binder compositions) in MB dye solution to evaluate their adsorption capacity and structural stability (Figures S9 and S10 attached in Supporting Information for 25 and 18 wt.% binder’s pellets, respectively).

### 2.3. Encapsulation of Activated Carbon

**Scheme 1:**
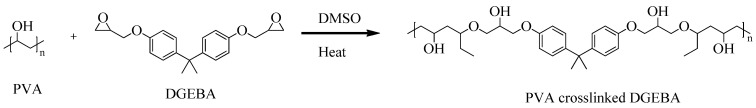
Crosslinking reaction of PVA with DGEBA in DMSO under heat, forming a stable polymer network.

The incorporation of AC particles into a crosslinked PVA-DGEBA matrix involved the formation of a three-dimensional (3D) polymer network, resulting in a solid, pelletized structure. As shown in Scheme 1, the hydroxyl groups of PVA chemically react with the epoxide functional groups of DGEBA, creating a covalently bonded 3D polymer network. The ground AC was homogeneously mixed into the transparent PVA-DGEBA blend, and the resulting mixture was then cast into molds and subjected to a stepwise thermal curing process, as schematically illustrated in Figure 1. This stepwise curing is defined as follows:

- 80 °C for 8 h: Initiates gelation and network formation.
- 120 °C for 4 h: Converts the gel into a more continuous polymer network.
- 160 °C for 4 h: Promotes further crosslinking and network stabilization.
- 190 °C for 8 h: Completes the curing process, ensuring a fully crosslinked, mechanically robust, and solvent-free pellet structure as explained by Figures S19 and S20 and Table S4 of EDS analysis.

This thermal process facilitates the progression of the crosslinking reaction, which was critical for achieving the desired mechanical strength, chemical stability, and structural integrity of the final pellets [27,41].

## 3. Results and Discussion

### 3.1. Surface Morphology Examination

**Figure 2:**
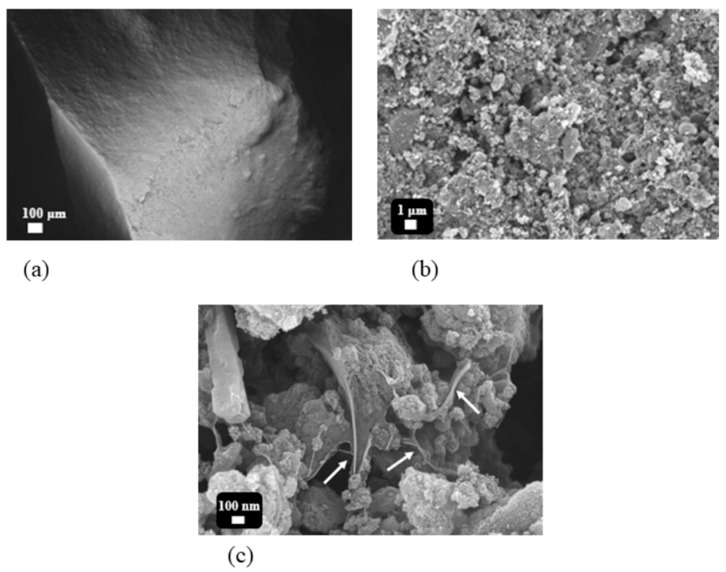
(**a**) FE-SEM image of AC pellet, (**b**) granular texture of AC particles in pellets, and (**c**) polymeric strands holding particles together (indicated by arrows).

The surface morphology of the prepared pellets was first examined to understand the effect of binder composition on the structural compactness of the AC particles. Field emission scanning electron microscopy (FE-SEM) images provide a stepwise visualization of the microstructure of the AC pellet containing 30 wt.% binder, as shown in Figure 2, progressing from a macroscopic to a microscopic perspective. At low magnification (100×, Figure 2a), the pellet surface appears smooth and densely packed, confirming successful pelletization and effective compaction of powdered AC into a well-defined structural form. In Figure 2b, granular textures and embedded AC particles become visible, demonstrating a homogeneous dispersion within the PVA–DGEBA polymer matrix. This uniform distribution indicates strong interfacial bonding and excellent cohesiveness of the binder, which contributes to the overall mechanical stability of the pellets. The higher-magnification image (Figure 2c) reveals the detailed morphology of the polymer binder network, where distinct polymeric strands or “bridges” formed by the crosslinked PVA–DGEBA matrix are observed encapsulating the AC particles. These bridges connect adjacent particles without excessively covering their surfaces, thereby maintaining the accessible porosity while ensuring structural reinforcement. In contrast, pellets with lower binder content exhibit less interconnection and appear more loosely stacked, leading to denser but mechanically weaker packing of particles, as shown in Figures S3 and S4 (Supporting Information). The clear visualization of these polymeric bridges provides compelling evidence of the binder’s critical role in reinforcing both the mechanical strength and the preservation of the functional integrity of the pellets.

### 3.2. FTIR and Thermal Analysis

**Figure 3:**
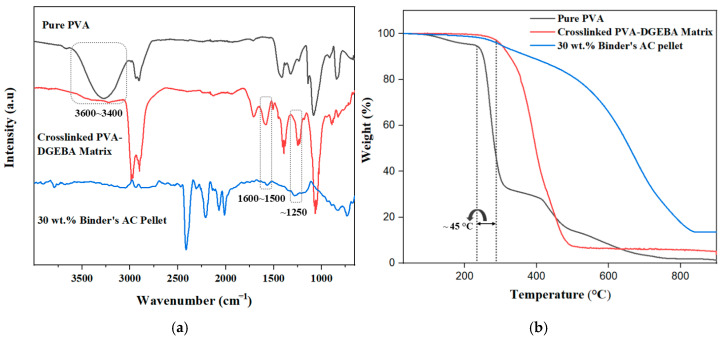
(**a**) FTIR spectrums of pure PVA, crosslinked PVA-DGEBA, and AC pellet. (**b**) Thermogravimetric analysis (TGA) curves of pure PVA, crosslinked PVA-DGEBA, and AC pellet.

Fourier transform infrared spectroscopy (FTIR) analysis was performed to confirm the successful crosslinking between PVA and DGEBA, as evidenced by distinct spectral modifications in Figure 3a. In the 3600–3400 cm^−1^ region, the broad O–H stretching band observed in pure PVA is reduced in the crosslinked PVA-DGEBA matrix, indicating the consumption of hydroxyl groups through reaction with epoxy groups to form crosslinked structures [42]. A small peak around 1700 cm^−1^ is attributed to carbonyl stretching vibrations from residual acetate groups, which is a known feature of incomplete hydrolyzed PVA [43]. The appearance of a pronounced peak near 1600–1500 cm^−1^ in the crosslinked sample confirms the presence of aromatic C=C stretching, reflecting the successful integration of DGEBA’s aromatic moieties into the polymer network [44]. Additionally, the peaks at approximately 1250 cm^−1^ and 1080 cm^−1^ correspond to C–O–C ether linkages [38] and the absence of the characteristic epoxy peak (typically found near 910–950 cm^−1^) in the crosslinked matrix also indicates near-complete reaction of the epoxy groups during the crosslinking process [44]. These features are also present in the 30 wt.% binder-based AC pellet, with small peaks in the 1700–1500 cm^−1^ region and signal near 1250 cm^−1^, confirming ether linkage formation and effective crosslinking within the pellet, thereby enhancing structural cohesion and environmental resistance. Together, these spectral features validate the formation of a crosslinked polymer network encapsulating AC particles. Notably, after crosslinking, the broad peak around 3300 cm^−1^ in the crosslinked matrix corresponds to O–H stretching, confirming the presence of hydrogen-bonded networks that facilitate electrostatic and hydrogen bonding interactions with MB molecules [45]. AC inherently contains surface hydroxyl (–OH) and carboxylic (–COOH) functional groups that also serve as the primary adsorption sites for MB [46] which can be confirm by observed adsorption. However, due to the strong infrared absorption nature of carbon materials, these bands often appear broad or weak in the FTIR spectrum [47].

Figure 3b illustrates the thermogravimetric analysis (TGA) of pure PVA, crosslinked PVA-DGEBA, and the 30 wt.% binder-based AC pellet, highlighting their distinct thermal degradation behaviors in the presence of nitrogen as a gas carrier. Pure PVA exhibited a rapid onset of thermal degradation at 240 °C; before this, the initial weight loss was attributed to moisture evaporation. This decomposition intensified sharply, with over 60% of the polymer mass lost by 320 °C, and continued until near-complete backbone degradation was observed around 650 °C [42]. In contrast, the crosslinked PVA-DGEBA matrix demonstrated enhanced thermal stability, with the onset of degradation delayed to approximately 285 °C. The degradation process was notably more gradual, indicating the effect of the crosslinked network in resisting thermal decomposition. The matrix continues to degrade steadily up to 500 °C [43,48]. The AC pellet containing 30 wt.% binder exhibited a similar initial degradation onset around 280 °C, corresponding to the decomposition of the polymeric binder. However, the weight loss progressed even more gradually, with significant mass retained beyond 500 °C [49,50]. The main activated carbon structure began to degrade after the binder and continued to decompose at around 820 °C. These findings clearly demonstrated the effect of crosslinking, highlighting the enhanced thermal stability observed in the crosslinked polymer matrix and polymer matrix-incorporated AC pellet, through their TGA curves compared to pure PVA.

### 3.3. X-Ray Diffraction Analysis

**Figure 4:**
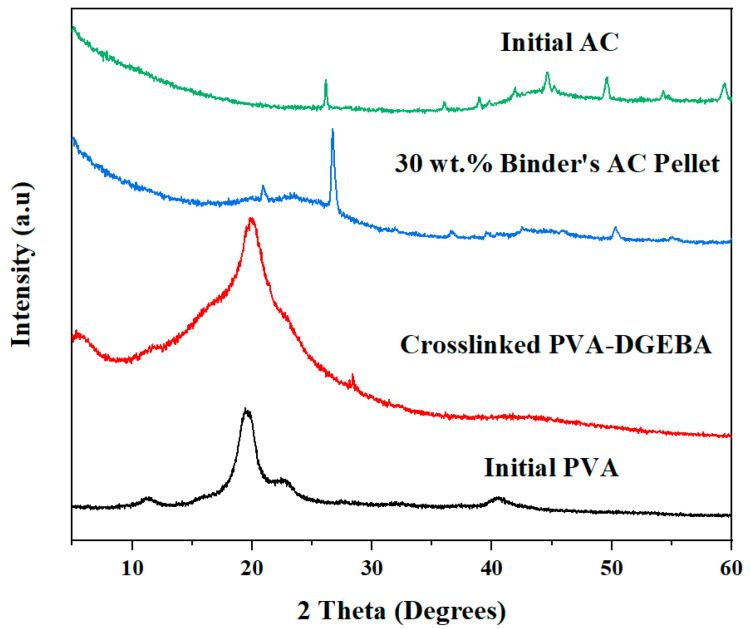
XRD spectrums of pure PVA, crosslinked PVA-DGEBA, 30 wt.% binder’s AC pellet, and initial AC.

The formation and structural integrity of the activated carbon (AC) pellets made by a crosslinked PVA-DGEBA polymer matrix were systematically verified through X-ray diffraction (XRD) analysis in Figure 4. The XRD pattern for pure PVA displays a sharp diffraction peak at around 2θ = 19.9°, a hallmark of PVA’s semicrystalline nature. Upon crosslinking with DGEBA, the primary diffraction peak of PVA becomes noticeably broader and less intense (Figure 4), indicating the disruption of PVA’s crystalline domains and an increase in amorphous character as the three-dimensional network forms. In addition, a weak and broad feature at approximately 2θ ≈ 6–8° appears in the crosslinked system. This low-angle reflection is attributed to short-range periodicity or microphase-separated domains, rather than a well-defined crystalline phase. Similar transitions have been reported for various crosslinked PVA systems, where crosslinking restricts chain mobility and leads to diminished crystallinity [38,51,52].

In Figure 4, the XRD spectrum of the AC pellet containing 30 wt.% PVA-DGEBA binder exhibits a distinctive composite fingerprint, featuring a broad peak at 2θ ≈ 20°, dominated by the crosslinked polymer binder, and a sharp peak at 2θ ≈ 26°, characteristic of the (002) reflection from microcrystalline or graphitic carbon [53], which was also revealed in the XRD of the initial activated carbon. The pronounced peak at 2θ ≈ 26°, set against a more diffuse background, highlights the partial retention of microcrystalline carbon domains from the activated carbon in the composite. Meanwhile, the underlying broad feature near 2θ ≈ 20°, attributed to the amorphous crosslinked PVA-DGEBA binder, as evidenced by comparison to the XRD of crosslinked PVA–DGEBA matrix reference, appears less intense due to its comparatively lower proportion and amorphous nature. This reduced intensity is consistent with the binder being finely and homogeneously dispersed within the porous activated carbon structure, rather than existing as large separate domains. This structural preservation of AC and uniform distribution of the binder maintain structural integrity to support the pellet’s adsorption and mechanical functions, demonstrating that the pellet is not merely a physical blend but a molecularly integrated and activated carbon composite [54,55].

### 3.4. Compressive Strength

Compressive strength is a crucial characteristic for evaluating the industrial viability of AC pellets, as it determines their ability to withstand harsh conditions and maintain structural integrity during prolonged use [10]. Table 1 below presents the mean compressive strength of three pellets for each composition, along with the standard deviations for each, highlighting a clear correlation between binder content and mechanical strength. Pellets with higher binder content exhibit significantly increased compressive strength, which is a relationship that is statistically supported by ANOVA analysis with post hoc comparisons using Tukey’s HSD test, as detailed in the Supplementary Information. These findings demonstrate that increased binder content enhances pellet durability, making them more suitable for industrial dye adsorption applications.

### 3.5. Swelling and Water Absorbance Analysis

Swelling ratios of pellets with varying binder content were determined using a gravimetric method [32]. The dried pellets were initially weighed and then immersed in water-filled vials for 12 h. After this soaking period, the pellets were removed, placed on blotting paper to eliminate surface moisture, and subsequently weighed to determine their final wet mass. The equilibrium swelling ratio (E_SR_) was calculated using the following equations:

(1) $$E_{SR\;(\%)=\;\frac{W_{t\;-\;\;W_{d}\;}}{W_{d}}\;\times 100}$$

where W_t_ is the weight of a wet pellet at a particular time (t); W_d_ is the weight of dry pellets before soaking.

For equilibrium water content,

(2) $$E_{WC\;(\%)=\frac{W_{e-\;W_{d\;}}}{W_{e}}\times 100}$$

where W_e_ is the weight of swollen pellets at equilibrium state.

Table 1 presents the equilibrium swelling ratio (E_SR_^a^) and equilibrium water content (E_WC_ ^b^) values of the pellets at saturation. Despite a significant increase in binder content from 18 wt.% to 30 wt.%, the equilibrium water uptake difference remains minimal, with only a 4% increment. This reduced water absorption in the 30 wt.% binder’s pellets highlights the effectiveness of superior crosslinking, resulting in fewer unreacted hydroxyl groups to attract water molecules. We also assessed their stability in different organic solvents through swelling and dissolution tests and found ~98% mass retention, and no deformation as shown in Figure S14 in the Supporting Information. These findings indicate that the 30 wt.% binder’s composition achieves higher mechanical strength and crosslinking efficiency, making it the most suitable candidate for further evaluation. Subsequent characterization tests will focus on this composition to verify the PVA-DGEBA crosslinking to encapsulate AC particles effectively and adsorption performance.

### 3.6. BET Analysis

**Figure 5:**
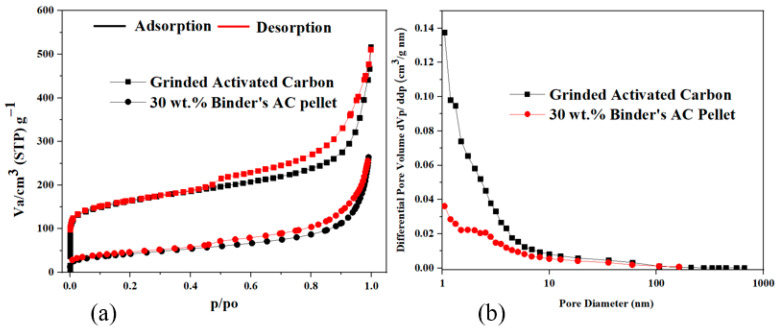
(**a**) Nitrogen adsorption–desorption isotherms of grinded AC and AC pellet measured at 77 K; (**b**) distribution of the pore size of grinded AC and AC pellet calculated using the BJH model.

The BET (Brunauer–Emmett–Teller) analysis provides key insights into the impact of binder incorporation (PVA and DGEBA) on the surface area and porosity of AC. Adsorption–desorption isotherms in Figure 5a reveal a clear distinction between grinded AC and the 30 wt.% binder’s pellets. The grinded AC exhibited a high adsorption volume (~500 cm^3^/g), reflecting its large surface area and highly porous structure. In contrast, the 30 wt.% binder’s pellet shows a lower adsorption volume (~250 cm^3^/g), indicating partial occlusion or blockage of pores, as confirmed by its Type IV isotherm with characteristic capillary condensation [56].

In addition, Figure 5b, which presents the pore size distribution of the materials calculated by the BJH method from the adsorption branch, provides a more comprehensive understanding of the structural changes caused by binder addition. The grinded AC displays a more concentrated pore size distribution, predominantly near to the microporous range (~2 nm), which aligns with its higher surface area and well-developed porosity. In contrast, the 30 wt.% binder’s pellets exhibited a broader distribution with a shift towards larger pore diameters (~2–50 nm), confirming the transition from a well-developed porous structure to restricted pores. This broader and flatter pore size distribution indicated that the binder addition leads to a reduction in the intensity of smaller pores, as evidenced by the diminished peak in the ~2 nm region. The relative increase in mesopores suggests that smaller pores are significantly affected during the pelletization process. This shift, together with the reduction in total pore volume, complements the findings from the adsorption–desorption isotherms (Figure 5a) and the summary in Table 2, further demonstrating the impact of binder incorporation on pore structure. Although binder incorporation reduces overall porosity, it enhances practical functionality by improving material recovery, handling efficiency, and environmental safety. Moreover, it introduces a low-temperature polymer crosslinking approach, enabling the formation of highly stable pellets while maintaining decent adsorption performance for methylene blue dye, making the pelletized form of AC a viable alternative for adsorption applications [57].

### 3.7. Adsorption Analysis

**Figure 6:**
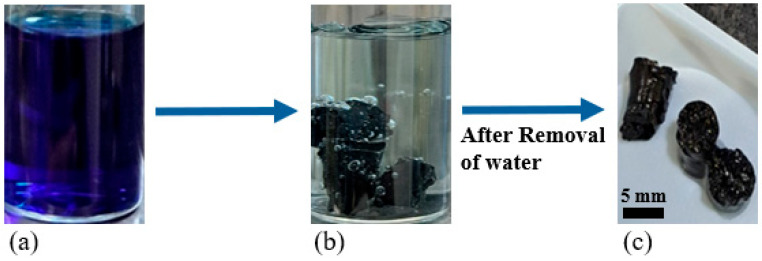
Optical images of (**a**) initial MB solution, (**b**) MB solution after around 2 days of AC pellet addition, and (**c**) MB solution with undissolved and strengthened AC pellets.

As reported in various studies, methylene blue (MB) dye poses one of the highest toxic threats to the environment, especially to marine life, due to its extensive use in coloring denim and other industrial applications in aqueous media [58,59]. Therefore, its safe disposal is extremely crucial. For this reason, MB dye was selected as the target pollutant for adsorption in this study. Figure 6 demonstrates the adsorption capability of 30 wt.% AC pellets for removing MB dye from aqueous solutions. Upon introducing the AC pellets into the MB dye solution, a noticeable color change from deep blue to a significantly lighter shade was observed over time, clearly indicating successful adsorption. This adsorption process occurs due to the affinity of MB molecules towards the AC surface, which is rich in extensive surface areas and diverse porous structures, providing ample adsorption sites. Multiple adsorption mechanisms contribute synergistically to the efficient dye removal observed. Key physical interactions involved were Van der Waals forces, π-π stacking interactions, hydrogen bonding, and electrostatic attractions. Van der Waals interactions stabilize MB molecules upon close contact with AC surfaces. The π-π stacking interaction specifically occur between aromatic rings of MB molecules and the aromatic graphitic domains of AC, substantially enhancing the adsorption capacity [60,61]. Additionally, hydrogen bonding and electrostatic attractions between positively charged MB cations and negatively charged oxygen-containing surface functional groups (e.g., hydroxyl and carboxyl groups) further reinforce the adsorption process. Thermodynamic studies consistently highlight the spontaneous and endothermic nature of methylene blue adsorption onto activated carbon-based absorbents, with higher temperatures enhancing dye uptake due to increased molecular mobility and activation of adsorption sites. This behavior confirms that, for our system, these interactions energetically favor dye retention on AC surfaces [45,62,63]. Moreover, adsorption equilibrium and kinetic analyses frequently suggest a multilayer adsorption model, where an initial monolayer is formed, followed by subsequent multilayer buildup, which is facilitated by the porous and highly structured nature of AC. Collectively, these well-established mechanisms ensure effective dye molecule capture and retention, leading to efficient and sustained removal of MB from aqueous solutions [64,65].

The 30 wt.% binder’s AC pellet underwent multiple cycles to assess the durability and maximum capability of MB dye uptake from an aqueous solution (Figure S6 in the Supporting Information). A 300 mg pellet was added to different concentrated MB dye aqueous solutions for each cycle, and after around two days with minimal manual agitation, impressive dye adsorption was observed. After each time, the pellets were removed from the solution, dried at 110 °C for two hours, and reused. Remarkably, the 30 wt.% pellets maintained their structural integrity through multiple adsorption cycles and achieved a maximum adsorption capacity of approximately q _max_ = 0.0148 g MB dye/g pellet (14.8 mg MB dye/g pellet), which is ~50% of the initial activated carbon’s adsorption capability (detailed in Supporting Information; Figure S11). Similar tests were performed with the 25 wt.% and 18 wt.% binders’ pellets (Figures S9 and S10 attached in Supporting Information) using 480 mg and 700 mg of each in 0.02 g/L MB dye solutions, respectively, which also showed effective adsorption. However, the 30 wt.% binder’s pellets exhibited optimal performance in terms of crosslinking and mechanical strength, justifying the optimization of this composition.

**Figure 7:**
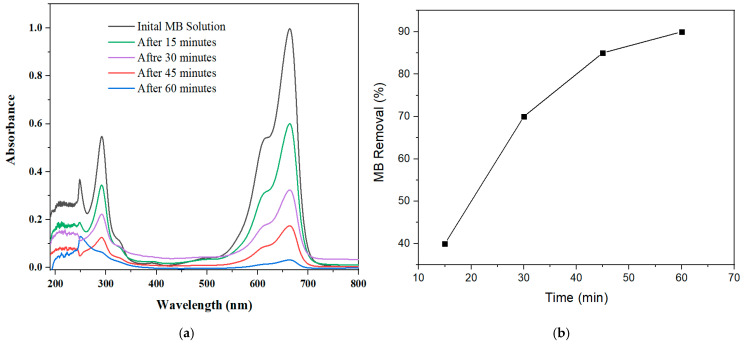
(**a**) UV–vis absorption spectra of methylene blue (MB) solution at different time intervals during adsorption using 30 wt.% binder’s AC pellet under magnetic stirring. (**b**) Corresponding MB removal efficiency (%) as a function of time.

We also evaluated the mechanical stability and adsorption efficiency of the 30 wt.% binder’s AC pellet under slightly rigorous conditions in the adsorption cycle to determine their industrial viability. A 75 mg AC pellet was immersed in a 0.025 g/L methylene blue (MB) aqueous solution and magnetically stirred at 200 rpm to enhance interactions between the pellet surface and MB molecules. As shown in Figure 7a, UV–visible absorbance monitoring revealed four initial characteristic MB peaks, with two in the UV region (244 nm and 290 nm) and two in the visible region (610 nm and 660 nm), corresponding to phenothiazine groups and aromatic chromophores [66]. Under stirring at room temperature, a significant decrease in peak intensities was observed progressively over time. Within the first 15 min, the highest absorbance peak at 660 nm dropped by approximately 40%, indicating rapid initial dye uptake. By 30 min, this peak’s intensity had further declined to about 30%, followed by a continued decrease to around 15% at 45 min. After 60 min, the absorbance peaks diminished under baseline levels (~10%), demonstrating near-complete MB removal. This time-dependent removal is summarized in Figure 7b, where MB removal efficiency increased steadily, reaching nearly 90% within one hour. This steady decrease in absorbance confirmed strong and sustained adsorption performance, while the pellets maintained their structural integrity throughout, demonstrating their promise as a scalable and cost-effective adsorbent for industrial use [67].

### 3.8. Adsorption Mechanisms: Isotherms, Kinetics, and Thermodynamics Studies

To further investigate the adsorption behavior of the AC pellets, adsorption models were applied and the corresponding kinetic, isotherm, and thermodynamic parameters were derived from the experimental data [68,69]. The detailed fitting procedures and equations are provided in the Supplementary Information, and the main kinetic, isotherm, and thermodynamic parameters are summarized in Table 3, Table 4 and Table S7.

#### 3.8.1. Kinetic Studies

Pseudo-first-order, pseudo-second-order, and intraparticle-diffusion models were used to analyze the MB uptake as a function of contact time. The pseudo-first-order model gave the best agreement with the experimental data, showing both the highest correlation coefficient and an equilibrium capacity very close to the measured value, which indicates that MB adsorption on the AC pellets is predominantly governed by a first-order process under the conditions studied (Table 3 and Supplementary Information).

#### 3.8.2. Adsorption Isotherm Studies

Equilibrium data were further interpreted using the Langmuir and Freundlich isotherm models. As summarized in Table 4, both models adequately describe the MB adsorption equilibrium, with the Langmuir model giving a slightly higher correlation coefficient, suggesting mainly monolayer adsorption on a relatively homogeneous surface under the present conditions. The fitting details are given in the Supplementary Information.

#### 3.8.3. Thermodynamic Studies

The temperature dependence of MB adsorption on the AC pellets was evaluated from equilibrium data collected at 50–80 °C. Equilibrium constants $K_{c}$ were obtained from the ratio of the amount of MB adsorbed to that remaining in the solution, and the standard Gibbs free energy change at each temperature was calculated using $\Delta G^{\circ }=-RTlnK_{c}$ (Table S7, Supplementary Information). A van’t Hoff plot of $\ln K_{c}$ versus 1/T produced a linear relationship, from which the thermodynamic parameters were determined as $\Delta H^{\circ }=+54.0$ kJ mol^−1^ and $\Delta S^{\circ }=+201$ J mol^−1^ K^−1^ (Supplementary Information).

These thermodynamic parameters indicate that MB adsorption on the AC pellets is spontaneous and endothermic under the studied conditions. Observing the negative $\Delta G^{\circ }$ values across the whole temperature range reveals that they became more increasingly negative with higher temperature, suggesting that higher temperatures thermodynamically favor dye uptake. The positive $\Delta H^{\circ }$ confirms the endothermic nature of the process, whereas the positive $\Delta S^{\circ }$ suggests increased disorder at the solid–solution interface, which is consistent with the slight enhancement in equilibrium adsorption capacity with temperature and with the mainly monolayer adsorption behavior inferred from the isotherm analysis.

## 4. Conclusions

Our primary objective was to mitigate pellet degradation under different environmental conditions in conventional pelletization methods and address the handling challenges associated with powdered AC on an industrial scale. This was achieved by converting powdered AC into stable cylindrical pellets through polymer crosslinking at low temperatures, while preserving their adsorption property. FE-SEM analysis revealed effective encapsulation of AC particles by visible polymer strands, retaining the initial structural integrity. The good mechanical strength (3.37 ± 0.46 MPa) and sustained stability of the 30 wt.% binder’s AC pellets were observed as they retained their shape across the adsorption cycle of harsh operational condition and in diverse organic solvents, underscoring their superior crosslinking efficiency. The improved thermal stability observed in both the crosslinked polymer matrix and their bonded AC pellet in comparison to PVA through TGA curves clearly indicates successful polymer crosslinking. FTIR and XRD analyses further supported this, revealing reduced hydroxyl absorption peaks (3200–3600 cm^−1^), characteristic aromatic C=C stretching vibrations (1600–1630 cm^−1^), prominent ether (C–O–C) linkage peaks (1050–1150 cm^−1^, 1250 cm^−1^), and disruption of PVA crystallinity through DGEBA addition, resulting in the formation of a robust, crosslinked polymer matrix that binds the AC particles effectively into pellets. BET and adsorption studies demonstrated an optimal balance between porosity and consistent adsorption performance over multiple cycles retained 50% of the adsorption capability of the initial AC. These findings highlight the 30 wt.% binder’s AC pellets as a highly promising, durable, and sustainable solution for industrial-scale MB removal. Additionally, this low-temperature stable pelletization approach sets the stage for the development of advanced, cutting-edge adsorption-based technologies.

## Figures and Tables

**Table 1:** Compressive strength and swelling analysis of pellets.

Compositions	Compressive Strength	E_SR_ ^a^	E_WC_ ^b^
30 wt.% Binder’s Pellets	3.37 ± 0.46 MPa	64%	39%
25 wt.% Binder’s Pellets	2.13 ± 0.60 MPa	62%	38%
18 wt.% Binder’s Pellets	1.71 ± 0.69 MPa	56%	35%

^a^ Equilibrium swelling ratio; ^b^ equilibrium water content.

**Table 2:** Textural properties of grinded activated carbon and 30 wt.% binder’s AC pellet obtained from nitrogen adsorption–desorption analysis.

Properties	Grinded Activated Carbon	30 wt.% Binder’s AC Pellet
BET Surface Area (m^2^/g)	590.5	154.2
Total Pore Volume (cm^3^/g)	0.698	0.403
Average Pore Diameter (nm)	2.36	5.23
Volume of N_2_ adsorbed (V_m_ cm^3^(STP) g^−1^)	135.6	35.44

**Table 3:** Summary of kinetic model parameters and their fit to experimental data.

Model	Equation	Parameters	Value	R^2^
Pseudo-first order	ln (q_e_ − q_t_) = ln q_e_ − k_1_ t	(q_e_) (exp)	3.24 mg·g^−1^	0.99
		(k_1_)	0.077 min^−1^	
Pseudo- second order	t/q_t_ = 1/(k_2_ q_e_^2^) +t/q_e_	(q_e_) (model)	5.10 mg·g^−1^	0.96
		(k_2_)	5.1 × 10^−3^ g·mg^−1^·min^−1^	
Intraparticle diffusion	q_t_ = k_id_ t^1/2^ + C	k_id_	0.45 mg·g^−1^·min^−1^^/2^	0.95
		C	−0.22 mg·g^−1^	

**Table 4:** Summary of parameters for Langmuir and Freundlich adsorption models.

Model	Equation	Parameter	Value	R^2^
Langmuir	$q_{e}=\frac{Q_{m}K_{L}C_{e}}{1+K_{L}C_{e}}$	$Q_{m}$(mg g^−1^)	9.68	0.85
		$K_{L}$(L mg^−1^)	0.41	
Freundlich	$q_{e}=K_{F}C_{e}^{1/n}$	$K_{F}$(mg g^−1^) (L mg^−1^)^1/n^	2.98	0.82
		n	2.13	

## Data Availability

The original contributions presented in this study are included in the article/Supplementary Material. Further inquiries can be directed to the corresponding author.

## Supplementary Materials

The following supporting information can be downloaded at: https://www.mdpi.com/article/10.3390/ma19010155/s1, References [1,10,11,12,13,57,68,69,70] are cited in the Supplementary Materials.
