# Supplementary material for: Polymer Crosslinked Activated Carbon Pellets for Dye Adsorption
Source: Materials (Basel). 2026 Jan 2;19(1):155. doi: 10.3390/ma19010155 (PMC12786828; doi:10.3390/ma19010155)
Supplement: Supplementary file 1 [file materials-19-00155-s001.zip › materials-4033761-supplementary.pdf]

Polymer Crosslinked Activated Carbon Pellets for Dye Adsorption

Muhammad Hadi, Sungho Yoon*

Department of Chemistry, Chung Ang University, 84 Heukseok-ro, Dongjak-gu, Seoul 06974, Republic of Korea

*Corresponding author email: sunghoyoon@cau.ac.kr

1. Ball Milling of Initial Activated Carbon

1.1 Initial Activated Carbon

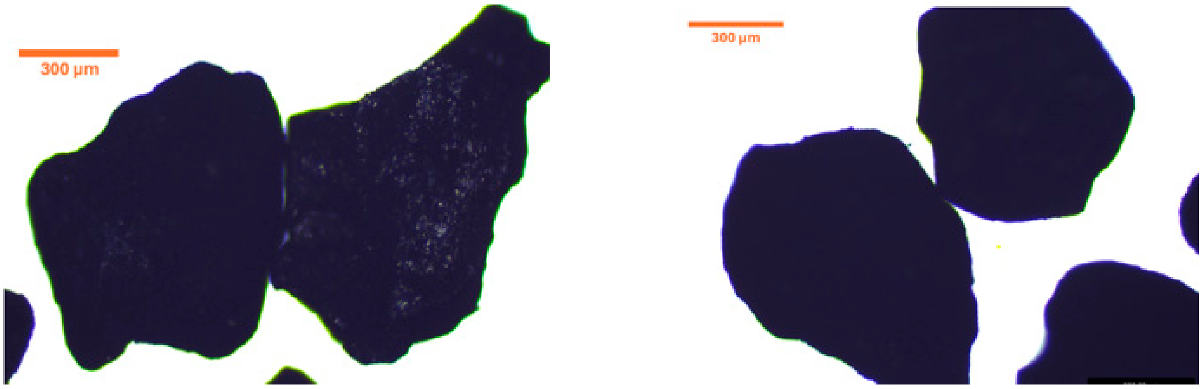

(a) (b)

Figure S1. (a) and (b) Company-Supplied activated carbon (AC) viewed under different micro scopic magnifications

As-received (Sigma) activated carbon (AC) with mesh size of + 420 to – 20. By microscope, we found the size range of around 500 to 800 μm.

1.2 Ball-Milled Activated Carbon

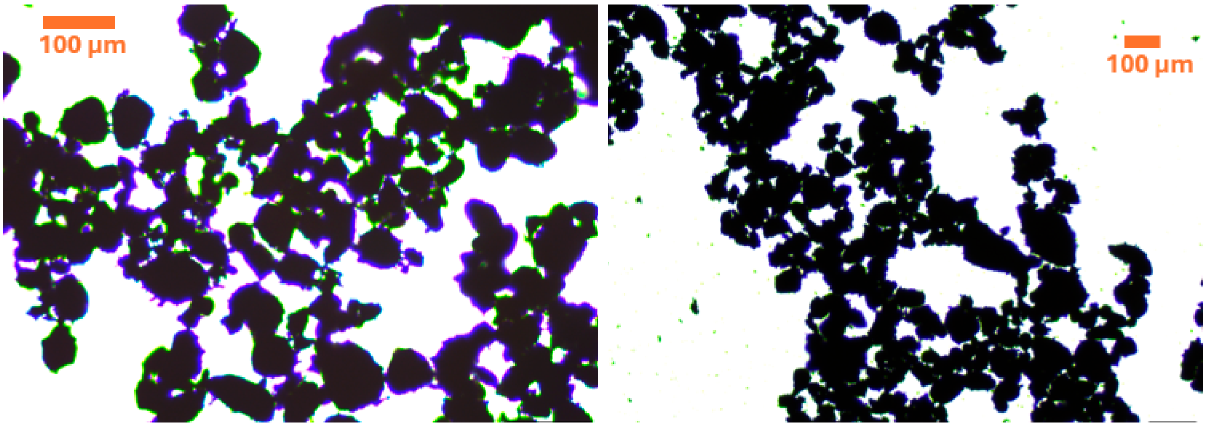

(a) (b)

Figure S2. (a) and (b) Ball-Milled Activated Carbon viewed under different microscopic mag nifications .

AC was grinded by a roller ball mill continuously for 12 hours, reducing the particle size und er 100 µm. It was analyzed by microscope.

2. FESEM of 18 and 25 wt.% Pellets with Initial Activated Carbon

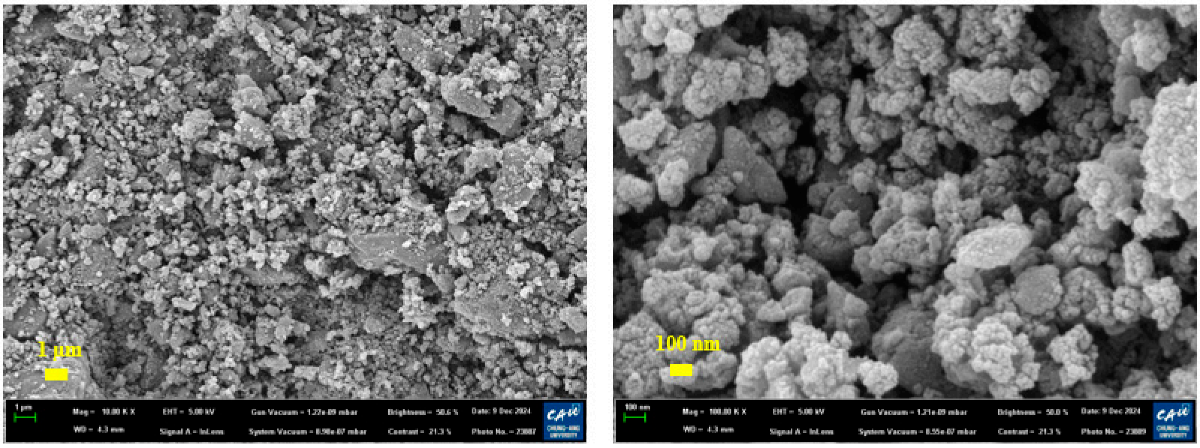

(a) (b)

Figure S3. (a) and (b) 18 wt.% binder's AC pellet at different magnifications.

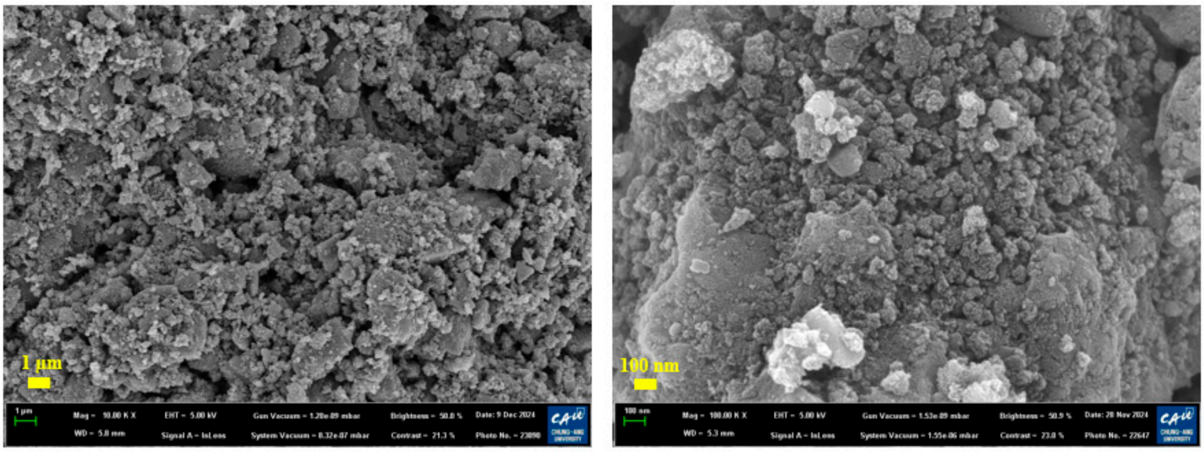

(a) (b)

Figure S4. (a) and (b) 25 wt.% binder's AC pellet at different magnifications.

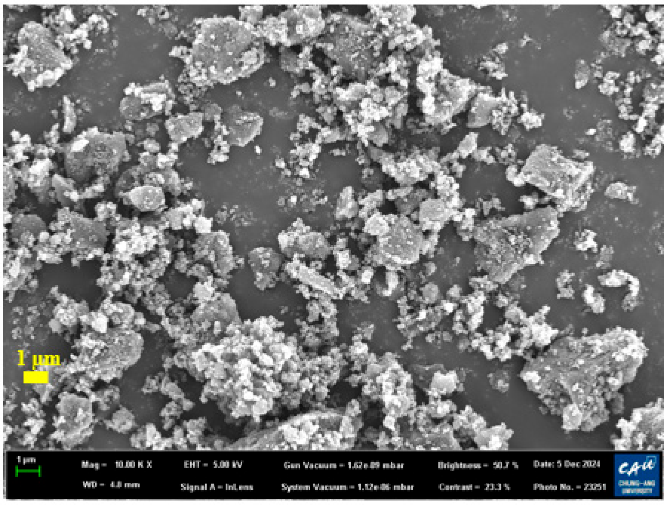

Figure S5. Initial activated carbon.

3. Repeated Methylene Blue Adsorption Using 30 wt.% Binder Pellets

A 300 mg AC pellet was subjected to five consecutive adsorption cycles in 12 mL MB solutions with initial concentrations of 60, 80, 100, 135, and 150 mg L^{-1}, respectively, with each contact lasting ~24 h under mild magnetic stirring to enhance mass transfer. The pellet exhibited efficient MB removal during the first four cycles, approaching its maximum adsorption capacity; in the fifth cycle, no appreciable additional uptake was observed, as illustrated in **Figure S6**.

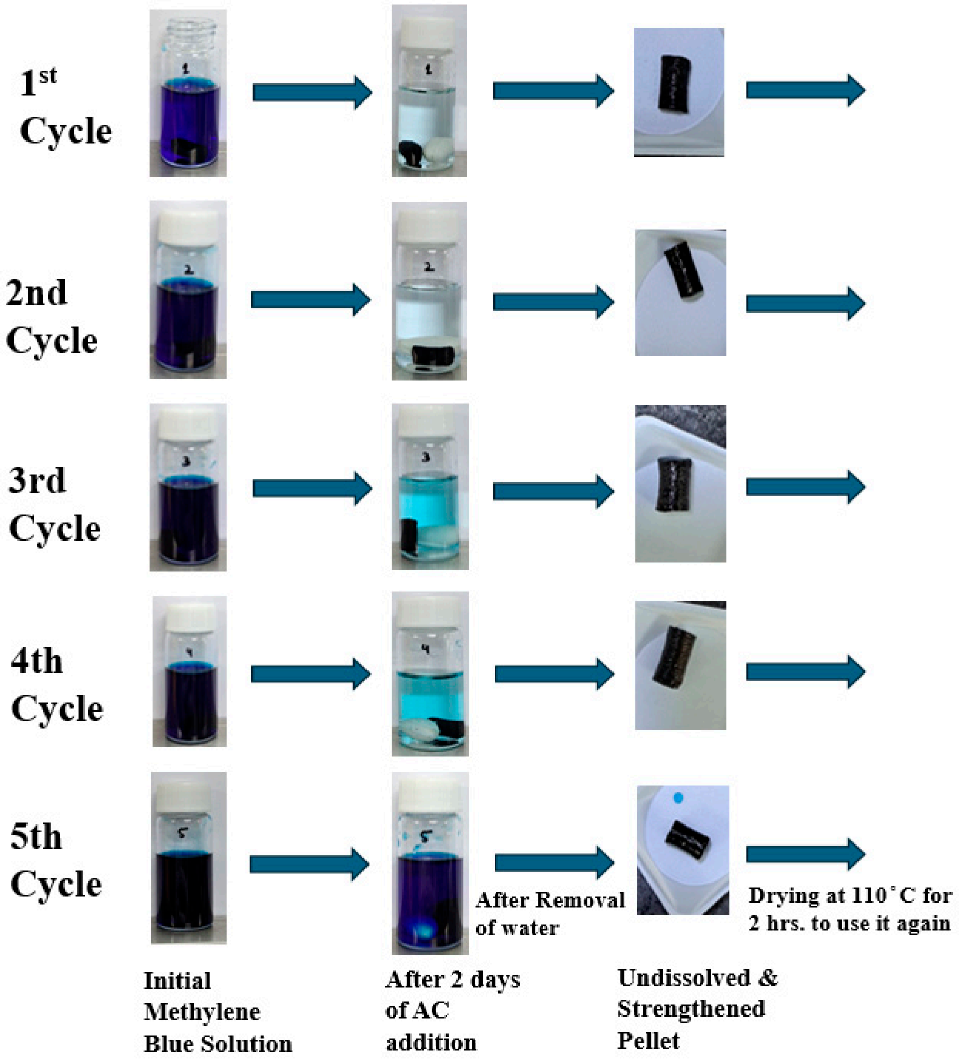

Figure S6. Optical images of the 30 wt.% AC pellet and MB solutions over successive adsorption–regeneration cycles.

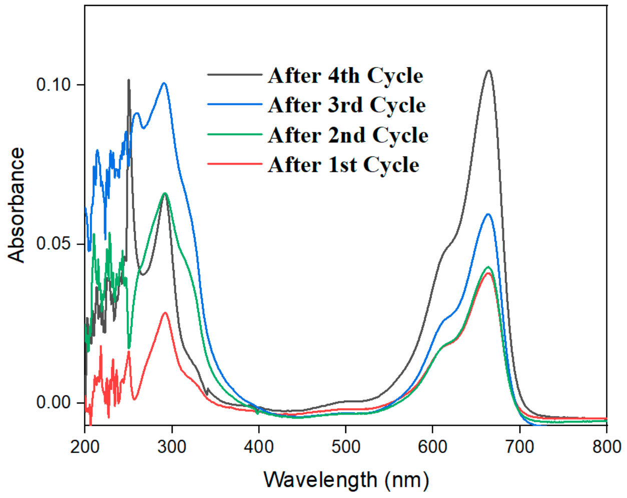

Figure S7. UV–vis spectra of the residual MB solutions after each adsorption cycle.

The residual MB concentration in each cycle was obtained from the UV–vis absorbance using the calibration curve (**Figure S7)**, giving values of ≈1 mg L^{-1} for the 1st and 2nd cycles, 1.2 mg L^{-1} for the 3rd cycle, and 2.2 mg L^{-1} for the 4th cycle (for initial concentrations of 60, 80, 100, and 135 mg L^{-1}, respectively). Converting these to the amount of MB adsorbed on the 300 mg pellet and summing over all cycles yields a cumulative adsorption capacity of about 14.8 mg g^{-1} as shown in **Figure S8**.

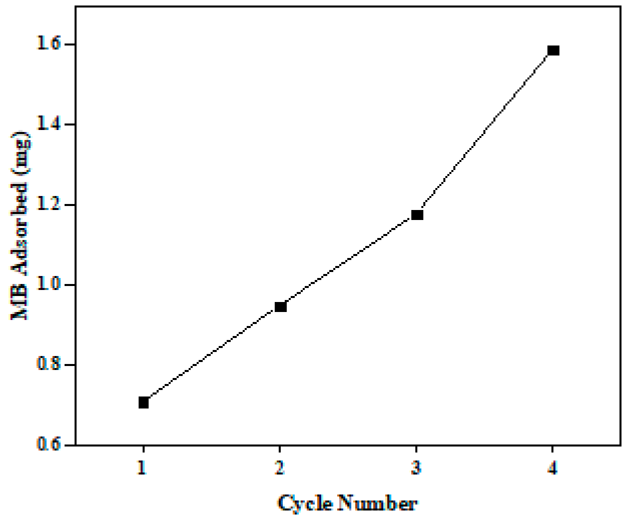

Figure S8. Variation in MB adsorption per cycle for the 30 wt.% AC pellet.

4. MB Dye Adsorption of the 25 wt.% Binder's Pellets

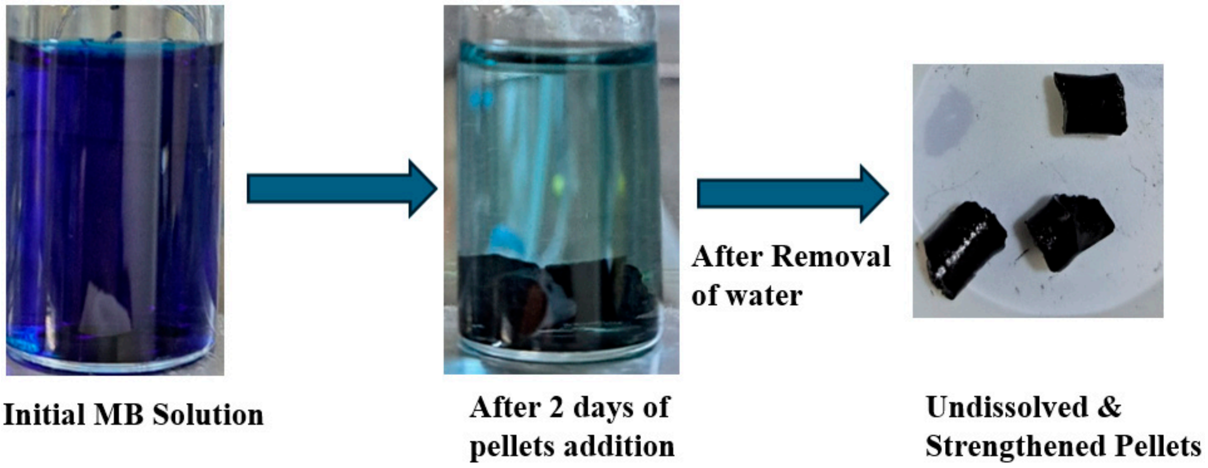

Figure S9. Adsorption of the 25 wt.% binder's pellets.

5. MB Dye Adsorption of 18 wt.% Binder's Pellets

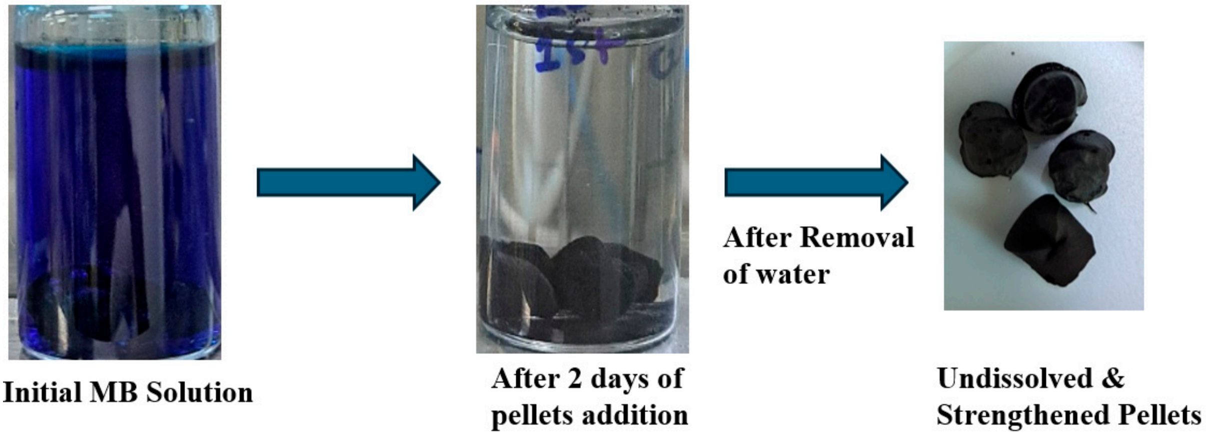

Figure S10. Adsorption of the 18 wt.% binder's pellets.

6. MB Adsorption Capability of Initial Activated Carbon

As shown in **Figure S11 and S12**, we conducted parallel MB adsorption tests using both PVA alone and AC alone to provide a direct comparison. For the AC experiment, 700 mg of activated carbon was applied in a 0.2 g/L (10 mg in 50 mL) MB solution for each cycle. The attached figure (**Figure S11)** demonstrates that AC efficiently removes MB during the first two cycles; however, its removal efficiency declines in subsequent cycles. By quantifying the total MB adsorbed (approximately 20 mg), the calculated adsorption capacity for our AC is 28.6 mg/g, as determined by **Equation S1** below. This value is lower than typical commercial powdered AC, which often possesses much higher BET surface areas (1000–3600 m²/g) [1], while our AC measures 496 m²/g, depending on origination and intended application.

$$q_e = \frac{Amount\ of\ MB\ Adsorbed\ (mg)}{Mass\ of\ AC\ (g)} \qquad \textbf{S1}$$

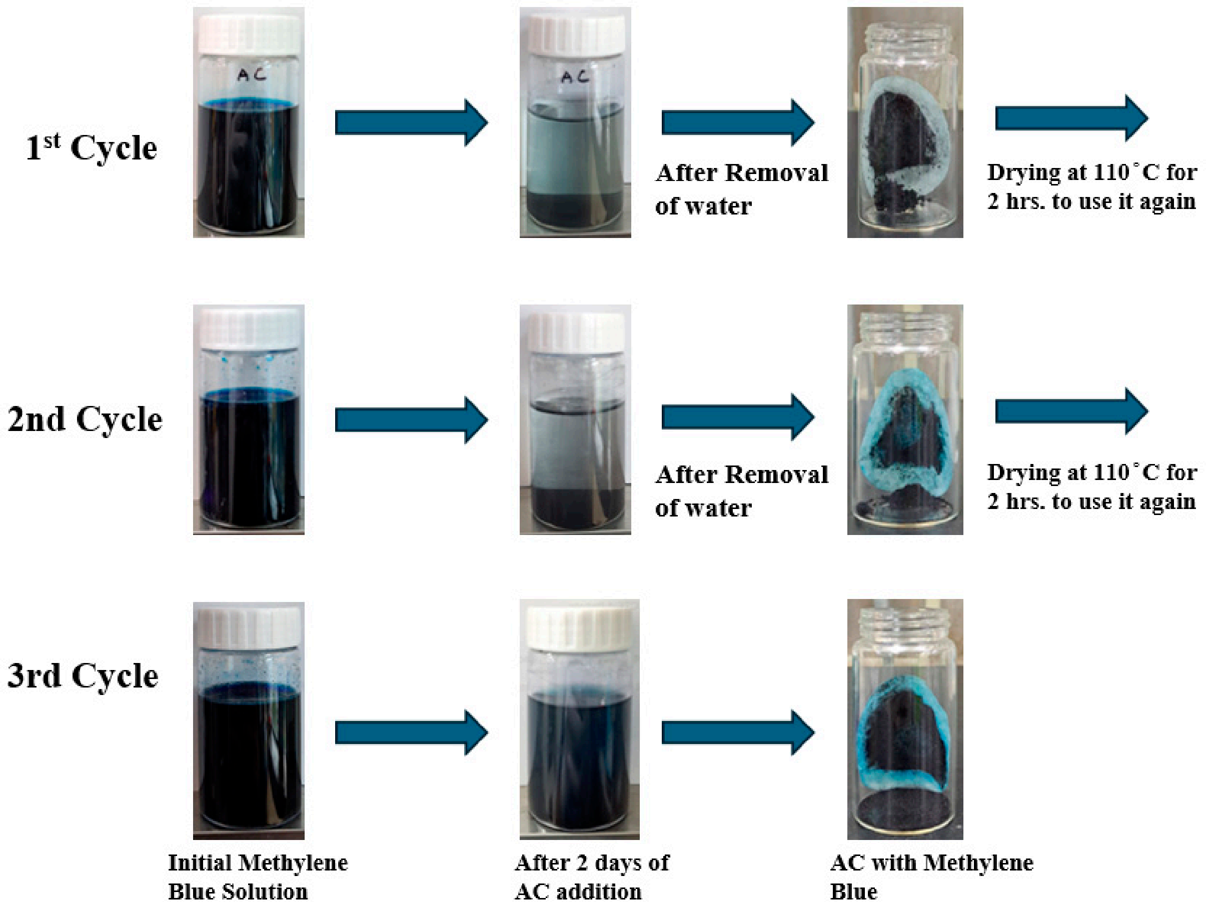

Figure S11. Adsorption capability of initial activated carbon.

7. Adsorption Capability of PVA

For PVA alone, 550 mg of PVA was tested in a 0.052 g/L MB solution (2.6 mg in 50 mL). No substantial MB adsorption was observed, as indicated by minimal color change in the s olution and only superficial blue coloration of the PVA sample. This reflects negligible re moval of MB from the solution and the absence of significant adsorption by PVA alone.

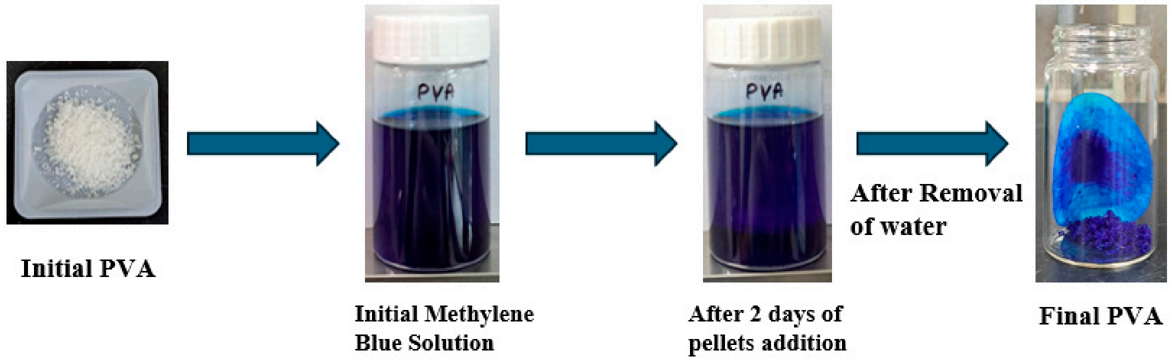

Figure S12. Adsorption capability of PVA.

8. Mechanical Strengths Analysis

We measured the hardness of three pellets with different binder compositions and calculated the standard deviation (SD), presenting the information in the table below. For compressive strength measurements, we recorded the diameter (~ 0.5 cm) of the circular surface where force was applied to break the pellets as shown in **Figure S13 (a).** The compressive strengths were then calculated and images of the pellets along with the hardness testing machine are provided for reference given below (**Figure S13) (b).** After calculating the compressive strength and SD, a one-way ANOVA was conducted to statistically evaluate the hardness differences among AC pellets with varying binder compositions. The analysis revealed a statistically significant difference in pellet hardness across the three binder levels ($F = 6.16$, $p = 0.035$), with data derived from **Table S1** given below. Post hoc comparisons using Tukey's HSD test showed that pellets containing 30 wt.% binder exhibited significantly greater hardness than those with 18 wt.% ($p = 0.032$). No statistically significant differences were observed between the 30 wt.% and 25 wt.% groups ($p = 0.135$), or between the 25 wt.% and 18 wt.% groups ($p = 0.513$).

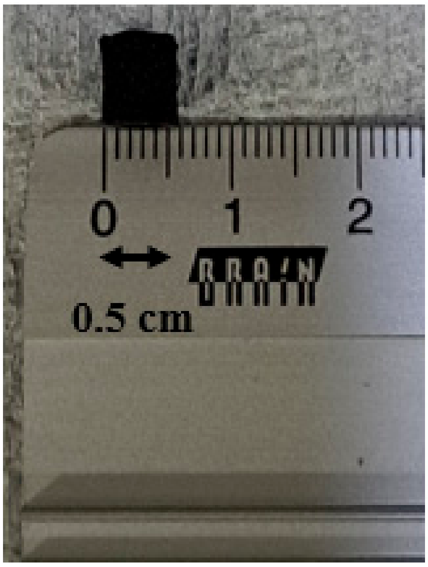

(a)

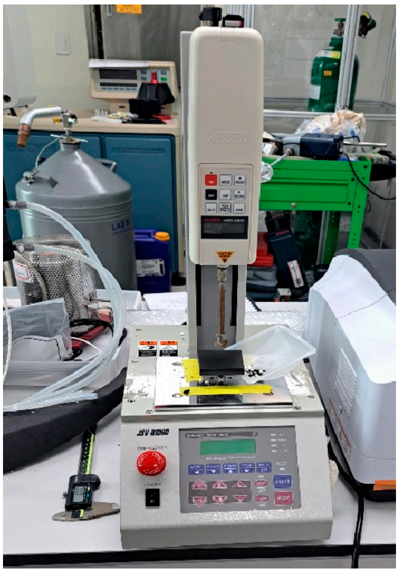

(b)

Figure S13. (a) Measurement of pellet's diameter and (b) hardness tester machine.

Table S1: Hardness (kg.F), mean values with standard deviations (kg.F), and compressive strength (MPa) for each pellet composition, categorized by binder content.

Composition		Hardness (kg.F)	Mean ± SD (Kg.F)	Mean ± SD (MPa)
30 wt.% Binder's Pellet	Pellet 1a	7.83	6.76±0.93	3.37 ± 0.46
	Pellet 1b	6.31		
	Pellet 1c	6.15		
25 wt.% Binder's Pellet	Pellet 2a	5.19	4.56±1.20	2.28 ± 0.60
	Pellet 2b	3.17		
	Pellet 2c	5.31		
18 wt.% Binder's Pellet	Pellet 3a	3.34	3.43±1.38	1.71 ± 0.69
	Pellet 3b	2.1		
	Pellet 3c	4.85		

9. Solvent Stability of the 30 wt.% AC Pellet

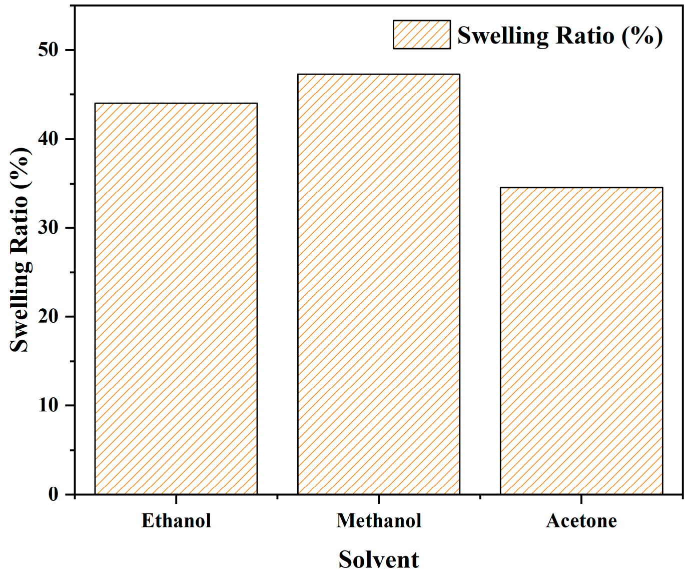

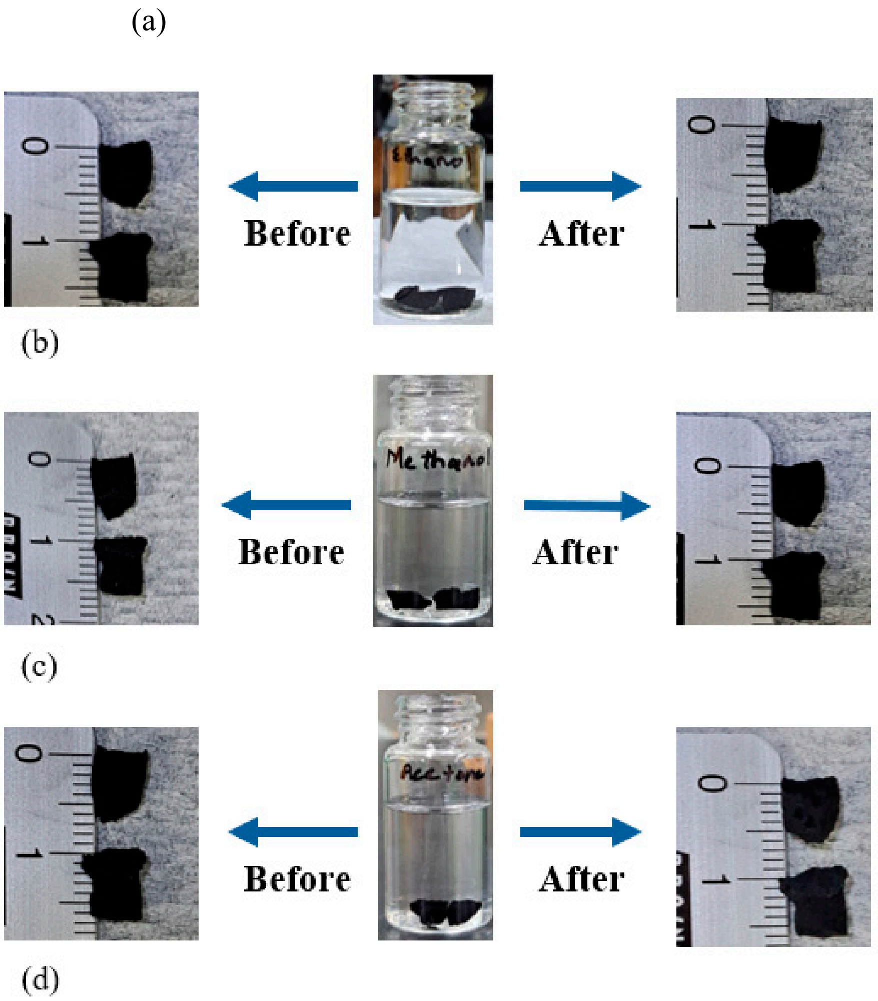

Figure S14. (a) Swelling ratio of pellets with different solvent and shape retention after eight-hour immersion in (b) ethanol, (c) methanol, and (d) acetone.

The 30 wt.% AC pellets were immersed separately in ethanol, methanol, and the aprotic solve nt acetone for 8 hours to evaluate their structural stability and swelling characteristics. Optical images, recorded alongside a scale bar, revealed excellent shape integrity with no visible defo rmation. Additionally, the mass retention was quantitatively assessed by weighing the pellets before swelling tests and after thorough drying post-immersion, confirming a high mass retent ion of approximately 98%, thereby demonstrating robust solvent resistance and structural dur ability.

10. Comparative Analysis of Classical Pelletization with Our Work

Table S2: Comparison of pelletization methods: highlights of our approach vs. the existing literature.

Pelletization Methodology/ Temperature (°C)	Pressure	Binder/Additives	Reference
Sintering (600-900)	380-890 MPa	PVA / PVP	[10]
Calcination (1100)	Not specified (extrusi on applied)	Clay (Kaolinite, qaurtz)	[11]
Sintering (800 - 840)	100- 500 MPa	None/ Ceramic oxide	[12]

Sintering (860-900)	245~735 MPa	None / Ceramic oxides	[13]
Sintering (560)	1.5 MPa	Sawdust	[57]
Sintering (850)	20 MPa	SCB	[70]
Crosslinking / Curing (190)	None	PVA-DGEBA	This work

Table S3: Comparison of dye adsorption performance of AC pellets from this work with reported pelletized activated carbons.

Adsorbent	Shape form	SA (m^2/g)	Percentage	Adsorbate	References
AC	Pellet	979	~79	Malachite Green Dye	[57]
HP-SCB-GA C	Pellet	804	~ 45	Methylene Blue Dye	[70]
AC	Pellet	150	~ 50	Methylene Blue Dye	This Work

11. Ball Milling Setup

We used a roller ball mill operating at 340 rpm continuously for 12 hours to grind the acti vated carbon and reduce its particle size. Zirconia balls of 28 mm in size diameter were us ed as the grinding media. To ensure transparency and reproducibility, the images of the ba ll milling setup is included below.

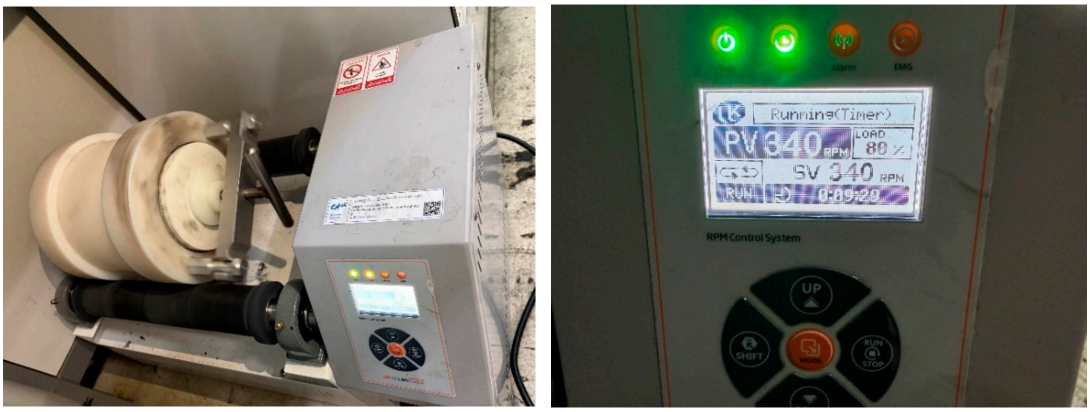

Figure S15. Ball milling setup for grinding activated carbon.

12. TGA Analysis of Initial Activated Carbon

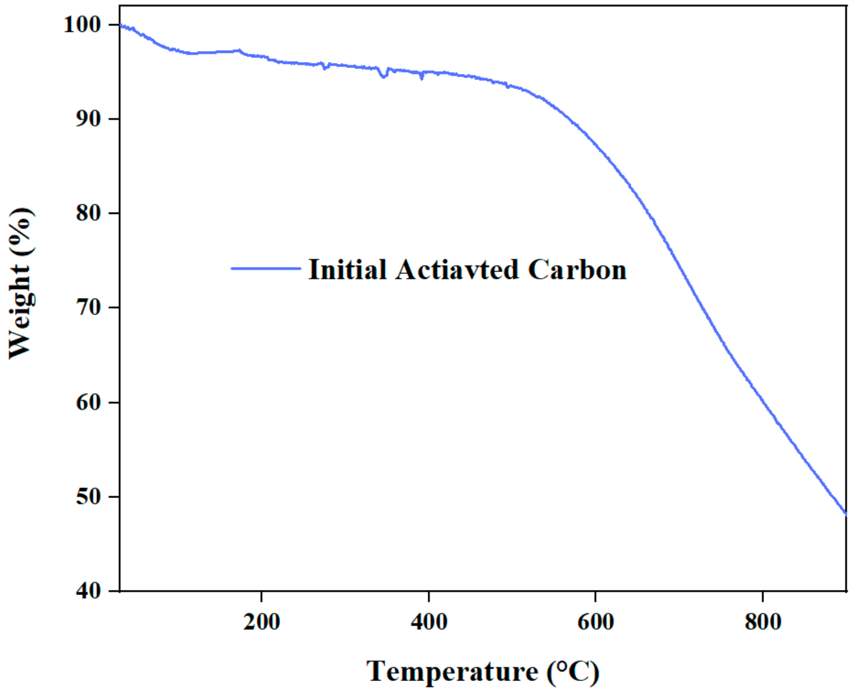

Figure S16. Thermogravimetric analysis of initial activated carbon.

13. Swelling Analysis of PVA-Alone AC Pellet

We prepared activated carbon (AC) pellets using 30 wt.% PVA as the sole binder (i.e., without DGEBA crosslinker), strictly adhering to the same methodology as described in the Experiment section of the revised manuscript. Swelling analyses were subsequently performed in several solvents, and the results are presented in bar graph as seen in **Figure S17** below. Consistent with the established literature, the PVA-alone AC pellets exhibit substantially higher swelling ratios across all solvents compared to the pellets fabricated with crosslinked PVA-DGEBA binder, even when the same amount of polymer content was used. This pronounced swelling is attributed to the abundance of uncrosslinked hydroxyl groups in linear PVA, which are highly hydrophilic and facilitate substantial solvent uptake, ultimately leading to rapid structural destabilization and fragmentation of the pellets upon immersion.

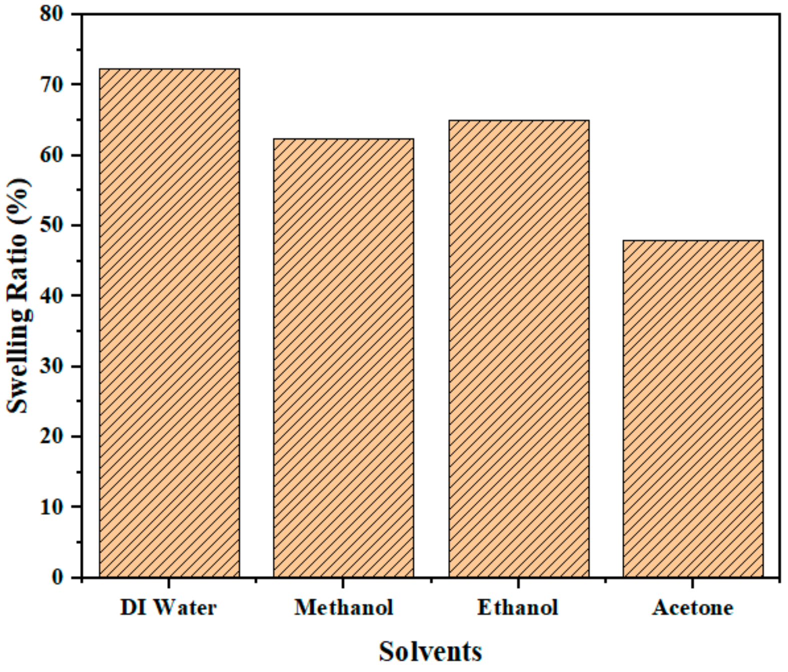

Figure S17. Swelling ratio of PVA-alone AC pellet.

14. TGA Analysis of PVA-Alone, 18, 25, and 30 wt.% Binder's AC Pellet

We conducted TGA analysis on AC pellets containing 18 wt.%, 25 wt.%, and 30 wt.% crosslinked binder, along with the PVA-alone AC pellet made by 30 wt.% of PVA. As shown in **Figure S18**, the results clearly demonstrate that pellets with the crosslinked PVA–DGEBA binder exhibit significantly greater thermal stability, characterized by higher decomposition onset temperatures and reduced weight loss up to 500 °C. Notably, the sample with 30 wt.% crosslinked binder shows the highest thermal resistance. In contrast, the PVA-alone AC pellet displays lower thermal stability, as evidenced by earlier and more rapid weight loss. This observation further supports our conclusion that the crosslinked binder system enhances the thermal stability of the AC pellets.

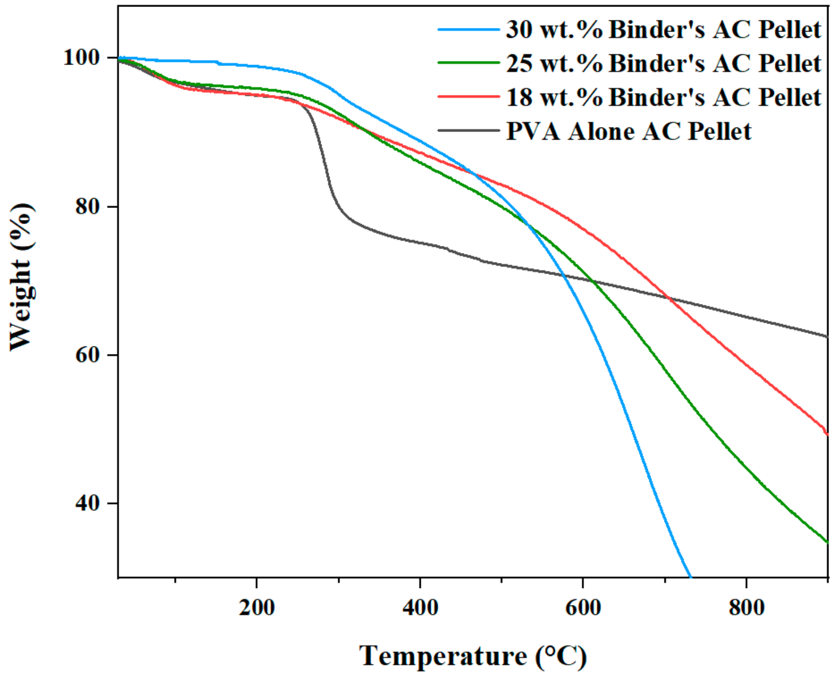

Figure S18. Thermogravimetric analysis of the PVA-alone AC pellet, and the 18, 25, and 30 wt.% binders' AC pellets.

15. EDS Analysis of Initial AC and the 30 wt.% Binder's AC Pellet

As we used DMSO in our pelletization process, we need to check the sulfur content to see whether our curing process has completely removed it or not because it can leach out while removing MB from water and contaminating it. So, we did the EDS analysis in this regard to check the amount of sulfur content in our 30 wt.% binder's AC pellet with initial AC as given below in **Figures S19, S20, and Table S4**. Notably, the oxygen content also increases significantly, which ensures the incorporation of the binder to create pellets.

EDS Analysis:

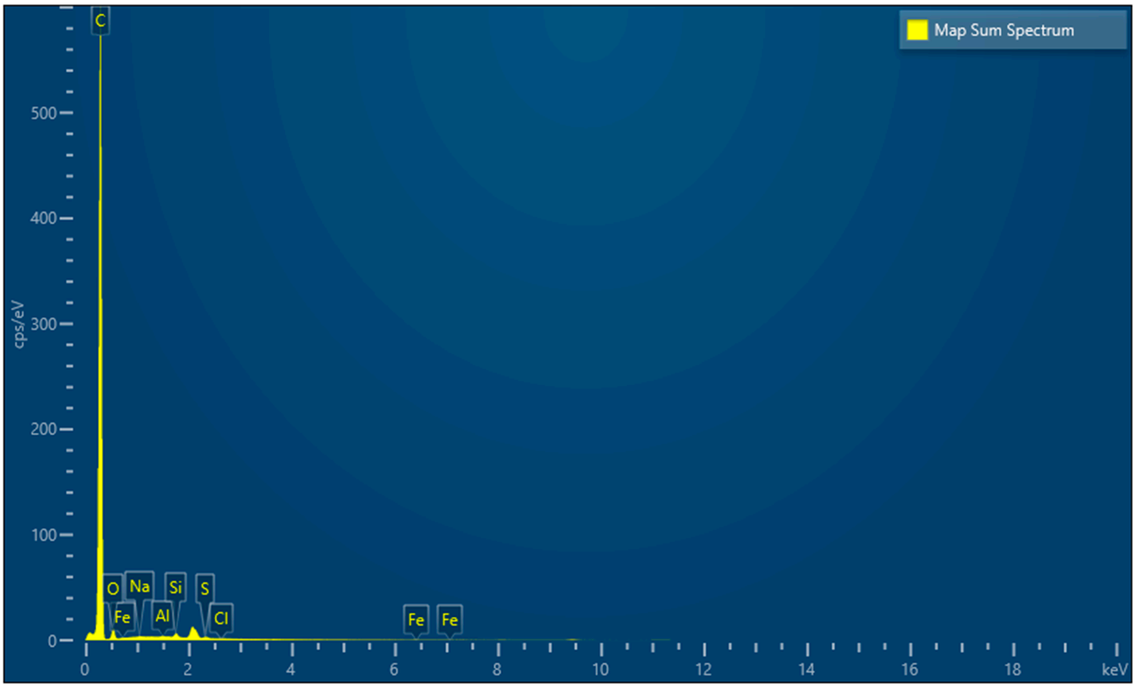

Figure S19. Spectrum of initial AC.

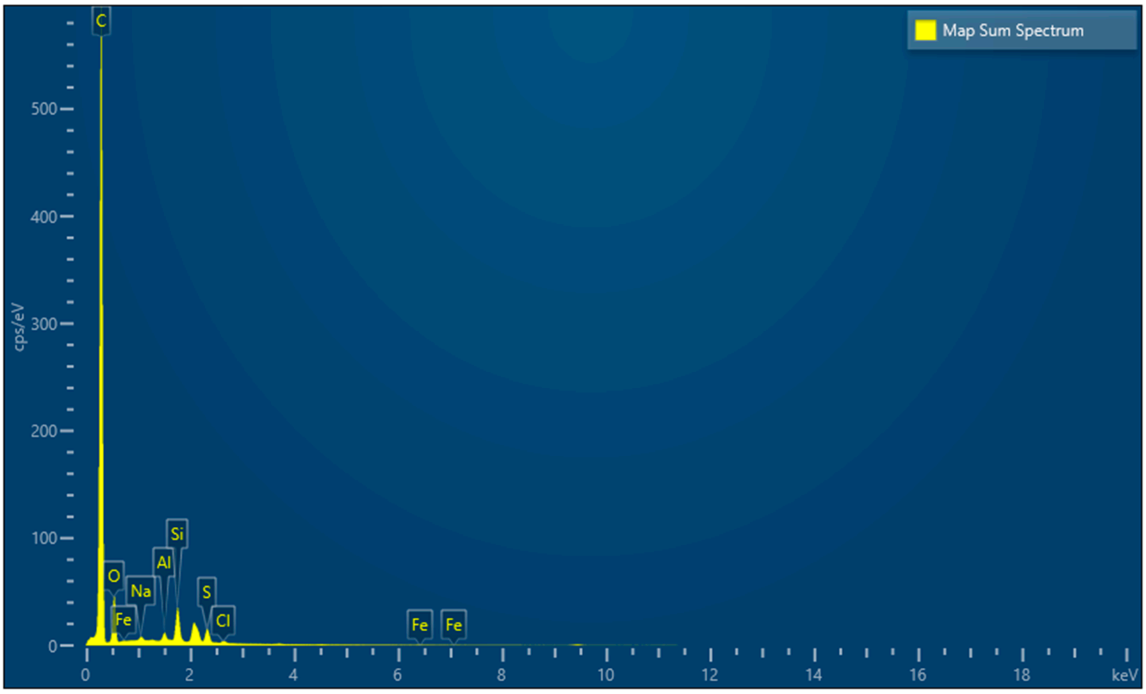

Figure S20. Spectrum of the 30 wt.% binder's AC pellet.

Table S4: EDS elemental analysis of initial AC and the 30 wt.% binder's AC pellet.

Quantitative Elemental Analysis										
**Elements**		C	O	Na	Al	Si	S	Cl	Fe	Total
**Quantity (wt.%)**	**Initial Activated Carbon**	94.72	4.77	0.04	0.06	0.24	0.1	0.01	0.07	100
	**30 wt.% Binder's AC Pellet**	84.55	12.66	0.19	0.33	1.35	0.76	0.14	0.03	100

16. Standard Calibration Curve for Methylene Blue Solution

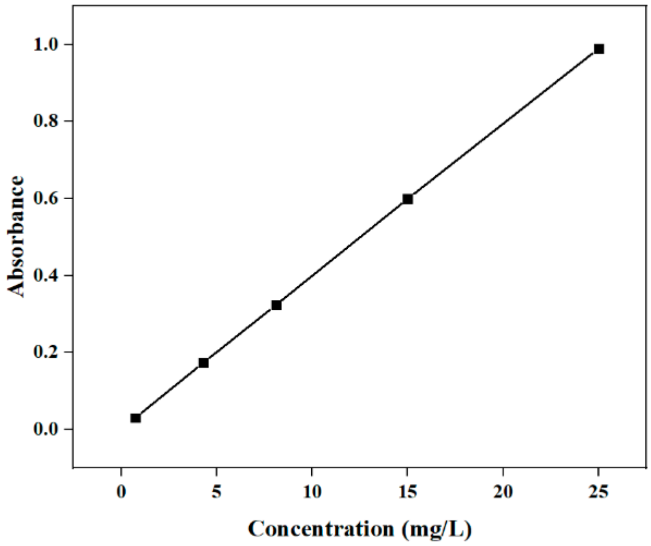

Figure S21. Standard calibration curve for methylene blue concentration in the solution.

17. Study of Adsorption Mechanisms: Isotherms, Kinetics, and Thermodynamics

17.1 Kinetic Studies

The concentration of methylene blue at each contact time was determined from the known initial concentration (25 mg L^{-1}, absorbance ≈ 1) using **Equation (S2)** and the UV–vis spectra in **Figure. (7a)**, as summarized in Table S5. The absorbance data in Figure 7. (a) was further used to construct a standard calibration curve at λ_{max} for the proceeding adsorption analysis [68],[69], as shown in **Figure S21**.

Firstly, C_t was calculated and then q_t:

$$C_t = C_0(A_t/A_o) \quad \textbf{S2}$$

$$q_t = \frac{(C_0 - C_t)V}{m} \quad \textbf{S3}$$

Table S5: Kinetic parameters for adsorption models at different time intervals.

t (min)	Absorbance (At)	C_t (mg/L)	q_t (mg/g)	q_e - q_t (mg/g)	ln(q_e-q_t)	t/q_t (min·g/mg)	√t ($min^{1/2}$)
15	0.599	15.0	1.33	1.91	0.65	11.3	3.873
30	0.323	8.10	2.25	0.99	−0.01	13.3	5.48
45	0.173	4.30	2.77	0.47	−0.75	16.2	6.71
60	0.030	0.75	3.24	0.0000	_	18.5	7.75

The equilibrium capacity was approximately 3.24 mg/g, determined from the final time point of the adsorption cycle at equilibrium.

$$q_e \approx 3.24\, mg/g$$

17.1.1 Pseudo-First Order (PFO) Kinetics

Model:

$$\ln\,(q_e - q_t) = \ln\, q_e - k_1 t \quad \textbf{S4}$$

Use t = 15, 30, 45 min (last point gives ln 0).

Regression of ln $(q_e - q_t)$ vs. t gives the following:

$$\ln\,(q_e - q_t) = 1.3575 - 0.077\, t$$

So,

$$k_1 = 0.077\, min^{-1}$$

$$R^2_{\mathrm{PFO}} = 0.99$$

This is an excellent fit for early-time data and gives a model qe very close to the experiment.

17.1.2 Pseudo-Second Order (PSO) Kinetics

Model:

$$\frac{t}{q_t} = \frac{1}{k_2 q_e^2} + \frac{t}{q_e} \quad \textbf{S5}$$

Linear regression of t/q_tvs t (all four points):

$$\frac{t}{q_t} = 7.40 + 0.196\,t$$

Hence,

$Slope = 0.196 = 1/q_e \rightarrow q_e^{\mathrm{PSO}} = 5.10$ mg/g

$Intercept = 7.40 = 1/(k_2 q_e^2) \rightarrow k_2 = 5.1 \times 10^{-3}$ g/mg.min

$$R_{\mathrm{PSO}}^2 = 0.964$$

Fit is good but predicted $q_e$is significantly higher than experimental 3.24 mg/g, and R^2 is worse than PFO.

17.1.3 Intraparticle Diffusion (Weber–Morris Model)

Model:

$$q_t = k_{id} t^{1/2} + C \qquad \textbf{S6}$$

Regression of $q_t$vs $\sqrt{t}$(all four points):

$$q_t = -0.22 + 0.45\,t^{1/2}$$

So,

$$k_{id} = 0.45\,mg.g^{-1}.min^{-1/2}$$

$C = -0.22\,mg.g^{-1}$ (non-zero → boundary-layer/film diffusion contributes)

$$R_{\mathrm{ID}}^2 = 0.95$$

Since the line does not pass through the origin and R^2 is lower than PFO, intraparticle diffusio n is not the only rate-controlling step.

17.2 Adsorption Isotherm

For the adsorption isotherm, the quantitative parameters for each of the four independent adsorption runs were summarized in **Table S6**. These values were obtained from the UV–vis analysis of the solutions after each run as shown in **Figure S22**, using the calibration curve shown in the corresponding figure (**Figure S21)**.

Table S6: Adsorption model parameters for various experimental runs.

Run	C_o (mg L^{-1})	C_e (mg L^{-1})	V (mL)	Pellet mass (mg)	q_e (mg g^{-1})
1	25	0.75	10	75	3.23
2	60	1.00	12	300	2.36
3	100	3.00	12	250	4.66
4	120	5.00	12	200	6.90

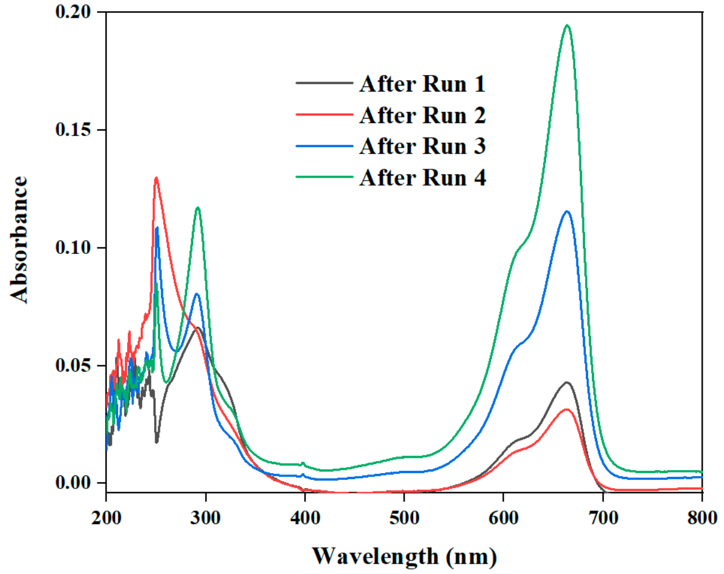

Figure S22. UV analysis of absorbance for remaining solution after each adsorption run.

q_e is calculated from $q_e = (C_0 - C_e)\, V/m$ as previously mentioned in **Equation S3**.

The equilibrium adsorption data were analyzed using the Langmuir and Freundlich isotherm models. The Langmuir model is given by the following:

$$q_e = \frac{Q_m K_L C_e}{1 + K_L C_e}, \qquad \textbf{S7}$$

which can be linearized as

$$\frac{C_e}{q_e} = \frac{1}{Q_m K_L} + \frac{C_e}{Q_m}.$$

A plot of C_e/q_e versus C_e afforded a straight line with slope $1/Q_m$ and intercept $1/(Q_m K_L)$. From this fit, the maximum monolayer adsorption capacity and Langmuir constant were determined to be $Q_m \approx 9.7$ mg g^{-1} and $K_L \approx 0.41$ L mg^{-1}, with a correlation coefficient $R^2 \approx 0.85$.

The Freundlich model is expressed as follows:

$$q_e = K_F C_e^{1/n}, \qquad \textbf{S8}$$

and its linear form is

$$\ln\, q_e = \ln\, K_F + \frac{1}{n} \ln\, C_e.$$

The linear regression of $\ln q_e$ versus $\ln C_e$ yielded $K_F \approx 3.0$ (mg g^{-1})(L mg^{-1})$^{1/n}$ and $n \approx 2.1$, with $R^2 \approx 0.82$.

Lastly, we summarized all the adsorption parameters in the Table 4 .

These results indicate that both Langmuir and Freundlich equations describe the equilibrium d ata reasonably well, with a slightly better fit for the Langmuir model under the present conditi ons.

17.3 Thermodynamic Studies

The thermodynamic behavior of MB adsorption on the AC pellets was evaluated from equilibrium experiments carried out at 50, 60, 70, and 80 °C, using the same initial MB solution and determining the residual equilibrium concentrations C_e from the UV–vis analysis shown in **Figure S23**, and standard calibration curve of **Figure S21**. The equilibrium constant K_c at each temperature was defined as the ratio of the amount of MB adsorbed to that remaining in the solution, and the corresponding standard Gibbs free energy change was calculated from **Equation (S9)** below.

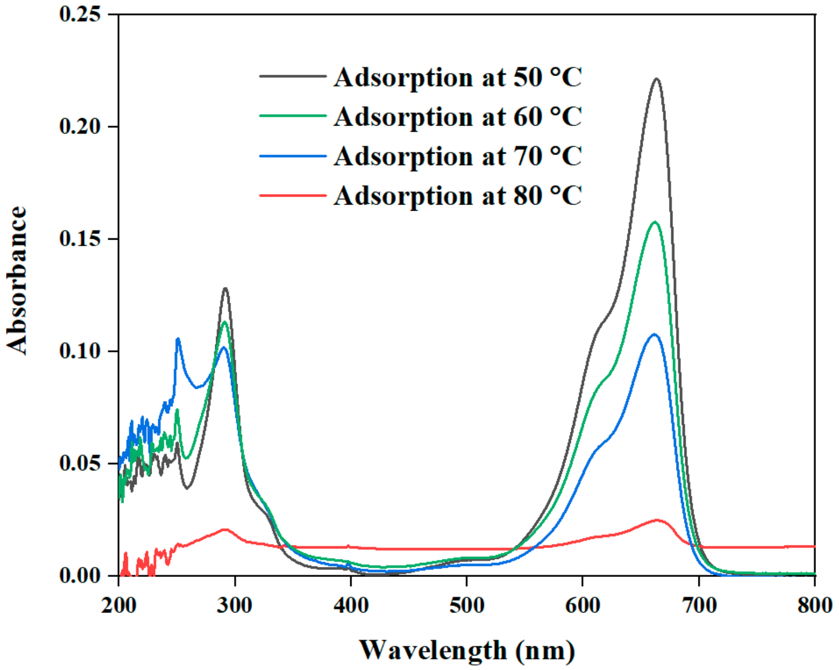

Figure S23. UV–vis spectra of the remaining MB solutions after adsorption at 50, 60, 70, and 80 °C.

Gibbs free energy:

$$\Delta G^\circ = -RT\ln K_c \qquad \textbf{S9}$$

where R is the universal gas constant (8.314 J mol^{-1} K^{-1}) and T is the absolute temperature (K). A van't Hoff plot of $\ln K_c$ versus $1/T$ was then used to determine the apparent enthalpy and entropy changes according to the following:

$$\ln K_c = -\frac{\Delta H^\circ}{R}\frac{1}{T} + \frac{\Delta S^\circ}{R} \qquad \textbf{S10}$$

so that the slope of the fitted straight line equals $-\Delta H^\circ/R$ and the intercept equals $\Delta S^\circ/R$.

The equilibrium concentrations, equilibrium constants, and Gibbs free energies obtained for each temperature are summarized in **Table S7**.

Table S7. Thermodynamic parameters for MB adsorption on AC pellets.

**Temperature (K)**	**1/T (K^{-1})**	**C_e(mg L^{-1})**	**K_c(dimensionless)**	**ln K_c**	**ΔG°(kJ mol^{-1})**
323	0.00309	5.5	69.0	4.23	−11.37
333	0.003003	3.9	97.6	4.58	−12.68

Temperature (K)	1/T (K^{-1})	C_e(mg L^{-1})	K_c(dimensionless)	ln K_c	$\Delta G°$(kJ mol^{-1})
343	0.002915	2.2	174.0	5.16	−14.71
353	0.002833	1.0	384.0	5.95	−17.46

The van't Hoff plot of ln K_c versus $1/T$ in the range 323–353 K yielded a straight line with a slope corresponding to $\Delta H° = +54.0$ kJ mol^{-1} and an intercept corresponding to $\Delta S° = +201$ J mol^{-1} K^{-1}. Since these parameters are obtained from a single linear fit, they are reported as constant over the studied temperature range, while $\Delta G°$ varies with temperature as listed in **Table S7**. The negative values of $\Delta G°$ at all temperatures reflect the spontaneous nature of MB adsorption on the AC pellets; the increasingly negative $\Delta G°$ with temperature indicates that adsorption becomes more thermodynamically favorable at higher temperatures. The positive $\Delta H°$ value confirms that the adsorption process is endothermic, and the large positive $\Delta S°$ suggests an increase in randomness at the solid–solution interface, consistent with the slight increase in equilibrium adsorption capacity with temperature and with the adsorption mechanism inferred from the kinetic and isotherm models.

18. Influence of Different Temperatures at Equilibrium Adsorption Capacity

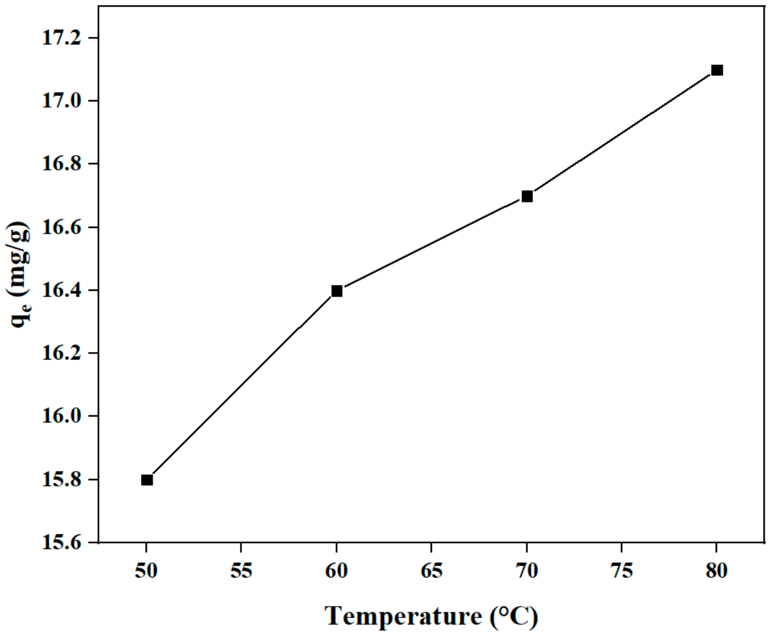

Figure S24. Effect of temperature on the equilibrium adsorption capacity qe of MB on AC pel -lets.

19. Characterization of the 30 wt.% Binder's AC Pellet After Multiple Adsorption Cycles

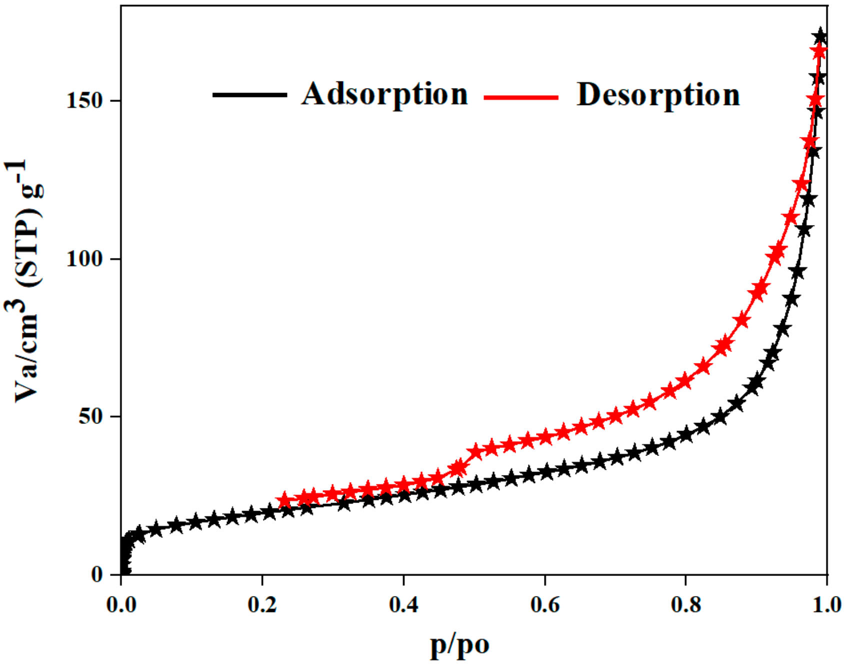

Figure S25. Nitrogen adsorption–desorption isotherms of AC pellet after multiple adsorption-s measured at 77 K.

Table S8. Textural properties of AC pellets after multiple adsorption cycles.

Properties	BET Surface Area (m^2/g)	Total Pore Volume (cm^3/g)	Average Pore Diameter (nm)	Volume of N_2 Adsorbed (V_m cm^3(STP) g^{-1})
	71.04	0.262	7.39	16.323

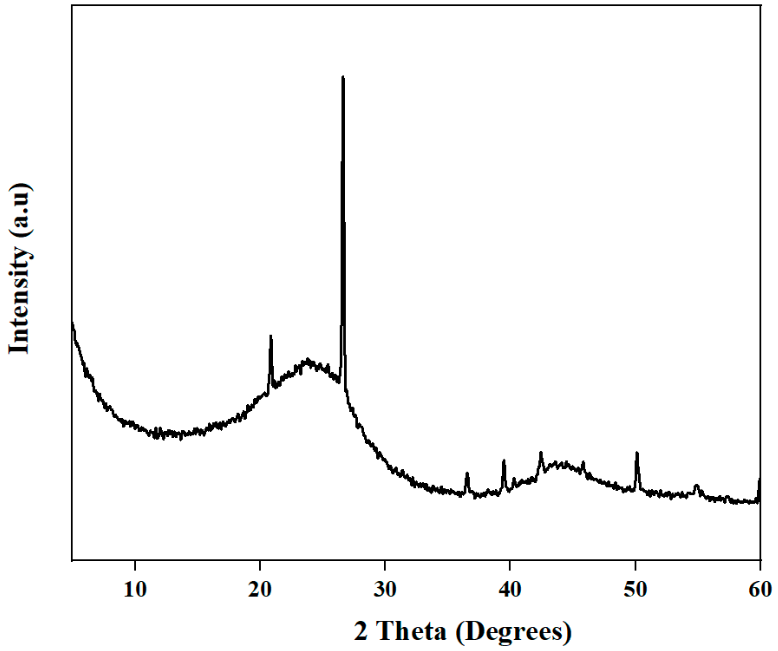

Figure S26. XRD analysis of AC pellet after multiple adsorptions.

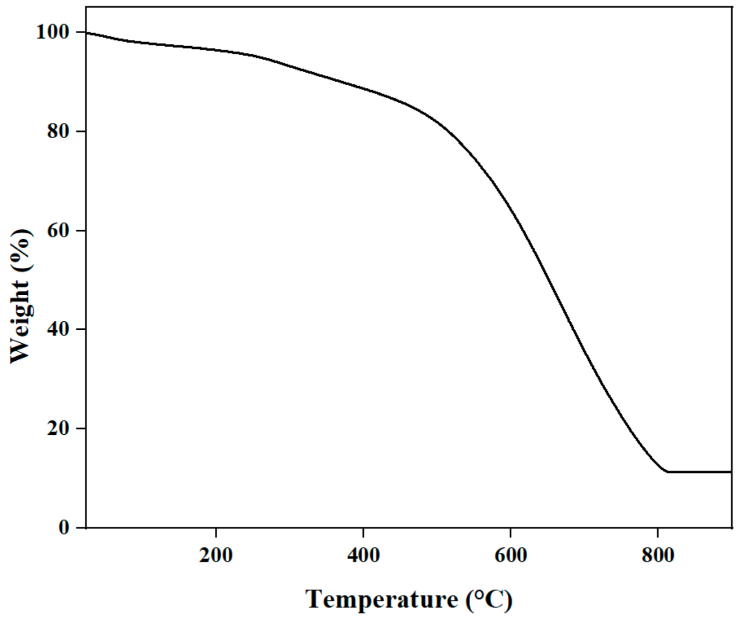

Figure S27. TGA analysis of AC pellet after multiple adsorptions.
